# A general approach to engineer positive-going eFRET voltage indicators

**DOI:** 10.1038/s41467-020-17322-1

**Published:** 2020-07-10

**Authors:** Ahmed S. Abdelfattah, Rosario Valenti, Jihong Zheng, Allan Wong, GENIE Project Team, Amy S. Chuong, Jeremy P. Hasseman, Vivek Jayaraman, Ilya Kolb, Wyatt Korff, Luke D. Lavis, Yajie Liang, Loren L. Looger, Derek Merryweather, Daniel Reep, Nelson Spruston, Karel Svoboda, Arthur Tsang, Getahun Tsegaye, Glenn Turner, Kaspar Podgorski, Minoru Koyama, Douglas S. Kim, Eric R. Schreiter

**Affiliations:** Janelia Research Campus, Howard Hughes Medical Institute, Ashburn, VA USA

**Keywords:** Fluorescence resonance energy transfer, Sensors and probes, Protein design, Neuroscience

## Abstract

Imaging membrane voltage from genetically defined cells offers the unique ability to report spatial and temporal dynamics of electrical signaling at cellular and circuit levels. Here, we present a general approach to engineer electrochromic fluorescence resonance energy transfer (eFRET) genetically encoded voltage indicators (GEVIs) with positive-going fluorescence response to membrane depolarization through rational manipulation of the native proton transport pathway in microbial rhodopsins. We transform the state-of-the-art eFRET GEVI Voltron into Positron, with kinetics and sensitivity equivalent to Voltron but flipped fluorescence signal polarity. We further apply this general approach to GEVIs containing different voltage sensitive rhodopsin domains and various fluorescent dye and fluorescent protein reporters.

## Abstract (web summary)

Genetically encoded voltage indicators (GEVIs) allow visualisation of fast action potentials in neurons but most are bright at rest and dimmer during an action potential. Here, the authors engineer electrochromic FRET GEVIs with fast, bright and positive-going fluorescence signals for in vivo imaging.

## Introduction

Genetically encoded voltage indicators (GEVIs) allow visualization of fast action potentials and subthreshold dynamics in groups of genetically defined neurons with high spatiotemporal resolution^1,2^. Monitoring voltage signals in vivo in large populations of neurons will enable dissection of detailed mechanistic links between brain activity and animal behavior. Despite recent advances, no current GEVI has ideal properties for routine, robust in vivo imaging. Knowledge of the mechanistic function of GEVIs will lead to improved designs. Existing GEVIs use voltage-sensitive protein domains from voltage-sensitive ion channels or phosphatases^3–7^ (VSD GEVIs), or microbial rhodopsin domains^8–14^ (rhodopsin GEVIs). Archaerhodopsin 3 (Arch) from ﻿*Halorubrum sodomense* was the first rhodopsin GEVI that accurately tracked changes in neuronal membrane potential^8^. However, Arch also generated a hyperpolarizing light-driven current upon exposure to imaging light^8,15^ by functioning as an outward proton pump^15^. A single amino acid substitution (D95N) in Arch abolished light-driven currents and retained Arch voltage-dependent fluorescence change^8^. The equivalent mutation in Ace1^12^, Ace2^12,14^, and Mac^13^ rhodopsins was used to generate later GEVIs.

The rhodopsin GEVI optical signal is fast and linear^8^, two desirable features for a voltage indicator. However, rhodopsin fluorescence is very dim, requiring intense illumination for imaging^8^. Fusions of fluorescent protein (FP) domains or other bright fluorophores to rhodopsin GEVIs were therefore made to facilitate imaging^11,13,14^. These fusions enable voltage-sensitive electrochromic fluorescence resonance energy transfer (eFRET) from a bright fluorophore to the retinal cofactor within the rhodopsin, which acts as a dark quencher. Although the absorbance of the retinal cofactor increases with increasing membrane potential, the emission of the eFRET-coupled fluorophore consequently decreases. All reported eFRET GEVIs therefore have negatively sloped fluorescence-voltage relationships; they are brighter at resting membrane potential and become dimmer during an action potential (negative going). Although two VSD GEVIs, FlicR^4^ and Marina^16^, exhibit positive-going signals in neurons, they have significantly slower response kinetics than eFRET GEVIs, making detection of action potentials difficult. Here we present a general approach to engineer eFRET GEVIs with fast, bright, and positive-going fluorescence signals in response to neuronal action potentials by modification of the natural proton transport pathway within microbial rhodopsins.

## Results

### Engineering a positive-going eFRET GEVI

**Fig. 1:**
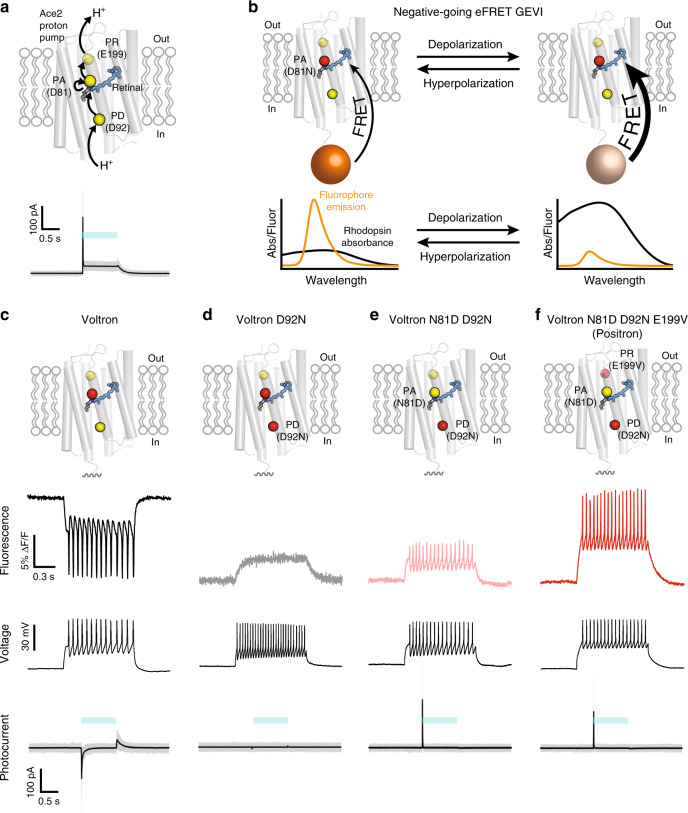
Engineering a positive-going rhodopsin eFRET GEVI. **a** Schematic (top) showing the hypothetical path of proton transport (arrows) through the Ace2 rhodopsin proton pump. The proton acceptor (PA), proton donor (PD), and proton release (PR) positions are represented as yellow spheres and labeled, retinal is shown as blue sticks. Photocurrent measurements (bottom) for the Ace2 rhodopsin proton pump. Steady-state photocurrent = 46 ± 11 pA (mean ± SD, *N* = 4 cells). **b** Schematic representing the proposed mechanism of negative-going rhodopsin eFRET GEVIs. Spectra^14^ in the lower panels have been scaled to illustrate the direction of absorbance and fluorescence change with membrane voltage change. We were not able to quantitatively measure these spectra in cell membranes at different voltages. At resting membrane potential, rhodopsin absorbance is low and therefore fluorophore emission is high. When the membrane depolarizes, rhodopsin absorbance increases leading to a decrease in fluorophore emission. **c**–**f** Schematic of amino acid substitutions (top), simultaneous fluorescence imaging (second row), and whole-cell patch-clamp membrane voltage measurements (third row), as well as photocurrent measurements (bottom) from rat hippocampal neurons in culture expressing Voltron, Voltron D92N, Voltron D92N N81D, and Positron labeled with JF_525_. Blue bar denotes time of light illumination ((508 nm–522 nm) at an irradiance of 70 mW mm^−2^) for photocurrent measurements. Steady-state photocurrents for all variants are negligible (−0.7 ± 3 pA, −0.1 ± 1 pA, 0.0 ± 1.5 pA, 0.1 ± 1 pA (mean ± SD, *N* = 5–7 cells), respectively). Simultaneous voltage and fluorescence traces are representative of *N* ≥ 3 cells.

Previous work fused the Ace2 rhodopsin from *Acetabularia acetabulum*^17^ to the FP mNeonGreen to produce a negative-going eFRET GEVI that allowed in vivo imaging of voltage signals in several model organisms^12^. We recently used the same Ace2 rhodopsin to engineer a negative-going chemigenetic eFRET GEVI called Voltron, which uses a HaloTag protein domain to covalently bind bright and photostable small-molecule flurophores^18,19^, extending the duration and number of neurons imaged simultaneously in vivo^14^. In both of these GEVIs, photocurrent of Ace2 rhodopsin (Fig. 1a) is blocked by mutating the residue that normally functions as the proton acceptor (PA)^20^ (D81N) (Fig. 1a, b), analogous to the Arch D95N mutation described above. This mutation blocks the primary pathway for exchange of protons from the retinal Schiff base, which links retinal to the rhodopsin protein, to outside the cell^20^. Electrophysiology measurements showing transient inward photocurrents with Ace2 D81N (Fig. 1c) and other rhodopsin-based GEVIs^21^, combined with previous mutagenesis and biochemical data^17,20^, suggest that voltage sensitivity in Ace2 D81N and other eFRET GEVIs results from membrane potential changes altering the equilibrium of protonation between the retinal Schiff base, the proton donor (PD) residue^20^, and the cell cytoplasm (Fig. 1a, b).

We hypothesized that we could alter the local electrochemical potential of protons on the retinal Schiff base and instead establish a protonation equilibrium with the outside of the cell, which would cause the rhodopsin absorbance and eFRET fluorescence to exhibit the opposite response to membrane potential change. Blocking access of protons to the retinal Schiff base from the cell cytoplasm should enable preferential exchange of protons with the outside of the cell. To block access of protons from the cytoplasmic side, we substituted the amino acid at the PD position of Voltron for a neutral residue (D92N). As expected, this substitution led to a block of the transient inward photocurrent of Voltron (Fig. 1d). Importantly, Voltron D92N (as well as other substitutions to neutral residues at the PD position (Supplementary Fig. 1)) showed a positive-going fluorescence signal with membrane depolarization, but with slow kinetics that made it incapable of following neuronal action potentials (Fig. 1d).

**Fig. 2:**
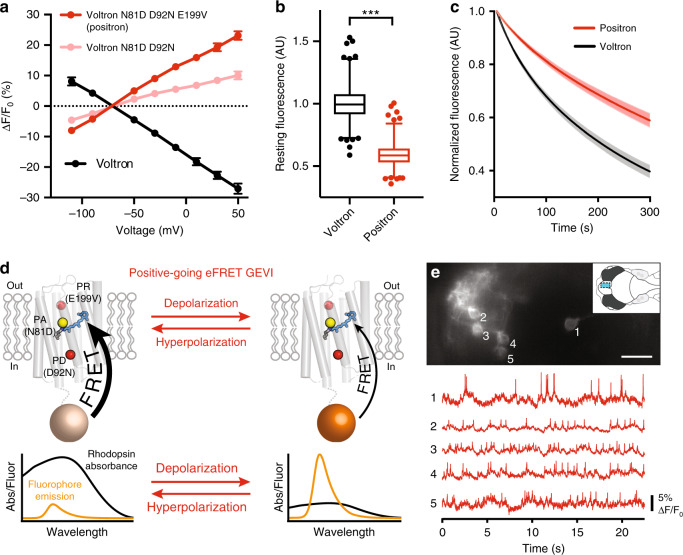
Characterization of positron neuron culture and larval zebrafish. **a** Graph of fluorescence vs. membrane voltage for Voltron (*N* = 6 cells), Voltron D92N N81D (*N* = 5 cells), and Positron (*N* = 5 cells) expressed in voltage-clamped rat hippocampal neurons in culture. Δ*F*/*F*_0_ measurements are mean ± SD. **b** Box and whisker plot showing the fluorescence of Voltron and Positron at resting membrane potential in neuron culture. Box represents interquartile range, center represents median, and whiskers represent 1st–99th percentile. Outliers shown as dots. *N* = 633 cells and 686 cells. ***P < 0.001 for two-tailed unpaired *t*-test. **c** Comparison of photobleaching of Positron and Voltron. Mean normalized fluorescence from a field of view of labeled, GEVI-expressing neurons is shown ±SEM. *N* = 19 fields of view each from *N* = 4 independent neuron cultures each. **d** Schematic representing the proposed mechanism of positive-going rhodopsin eFRET GEVIs. Spectra^14^ in the lower panels have been scaled to illustrate the direction of absorbance and fluorescence change with membrane voltage change. We were not able to quantitatively measure these spectra in cell membranes at different voltages. At resting membrane potential, rhodopsin absorbance is high and therefore fluorophore emission is quenched. When the membrane depolarizes, rhodopsin absorbance decreases leading to an increase in fluorophore emission. **e** Imaging of voltage signals from five neurons in the forebrain of live larval zebrafish. (top) Imaged field of view showing fluorescence from five individual neurons expressing Positron and labeled with JF_525_. Inset shows an overview of the larval zebrafish brain and the imaged area (blue rectangle). (bottom) Fluorescence of the five visible neurons over time. Scale bar: 20 μm Data are representative of *N* = 3 fish.

We reasoned that the proton pathway between the retinal Schiff base of Voltron D92N and the exterior of the cell was inefficient, resulting in the observed slow kinetics. To improve the efficiency of proton movement towards the outside of the cell, we substituted the amino acid at the PA position for a negatively charged aspartate (N81D), as was present in the original Ace2 rhodopsin sequence. This resulted in an indicator (Voltron N81D D92N) that had sufficient response speed to track action potentials in neurons (Fig. 1e). Critically, Voltron N81D D92N exhibited no steady-state photocurrent (Fig. 1e), showing that it can function as a GEVI without pumping protons across the membrane. Voltron N81D D92N had a transient outward photocurrent (Fig. 1e), confirming that the Schiff base proton was now in equilibrium with the outside of the cell. Although Voltron N81D D92N was suitable for monitoring neuronal action potentials with positive-going fluorescence changes, it showed only ~40% of the fluorescence change of the negative-going Voltron (Figs. 1e and 2a).

To improve the sensitivity of Voltron N81D D92N we focused on the rest of the proton transport pathway of the Ace2 rhodopsin. We reasoned that proton release (PR) residues^20^ near the outside of the cell should play a critical role in proton movement towards the cell exterior and possibly modulate the protonation equilibrium of the retinal Schiff base. Saturation mutagenesis of the two PR sites (positions 189 and 199 in Ace2) led to a variant with the substitution E199V that had ~2-fold improved dynamic range over Voltron N81D D92N (Fig. 1f, Fig. 2a, and Supplementary Fig. 2). We decided to name Voltron N81D D92N E199V as Positron due to its positive-going response to membrane depolarization. Compared with Voltron, Positron bound to the fluorescent dye JF_525_ had similar fluorescence response to voltage steps, but with a positive fluorescence-voltage slope (Fig. 2a and Supplementary Fig. 3). With sub-millisecond on and off time constants (Supplementary Fig. 4 and Supplementary Table 1), Positron clearly reported action potentials in neurons (Fig. 1f) with sensitivity equivalent to that of Voltron. Membrane trafficking of Positron was indistinguishable from Voltron (Supplementary Figs. 5 and 6), and expression of Positron did not alter the electrophysiological parameters of neurons in culture (Supplementary Fig. 7). We observed that Positron was 41% dimmer than Voltron at resting membrane potential in neurons (Fig. 2b) and bleached at approximately half the rate of Voltron (Fig. 2c), consistent with the hypothesis that at rest, the retinal Schiff base of Positron is more protonated and has a higher absorption, resulting in more eFRET and less dye fluorescence emission. When the membrane depolarizes, the proton is driven toward the outside of the cell, resulting in lower retinal absorption, less quenching of dye fluorescence, and a positive-going fluorescence response (Fig. 2d).

### Using Positron for in vivo voltage imaging

We previously showed that Voltron was suitable for imaging voltage signals in the brains of live animals such as fruit flies, zebrafish, and mice^14^. To confirm that Positron allows for in vivo imaging, we recorded optical voltage signals from five neurons simultaneously in the forebrain of a larval zebrafish expressing Positron and labeled with JF_525_ using a wide-field fluorescence microscope (Fig. 2e). We observed independent spiking and subthreshold signals in each of the neurons (Fig. 2e) with signals similar to those observed with Voltron previously in the same preparation (compare Fig. 2e with Fig. S34 of ref. ^14^). We also directly compared Positron and Voltron imaging of spontaneous action potential spikes in olfactory sensory neurons (OSNs) of larval zebrafish (Supplementary Fig. 8). Positron and Voltron provided equivalent SNR in this preparation and each showed robust signals at the end of the five-minute imaging window, suggesting that imaging could likely continue for significantly longer. We have only performed one-photon in vivo imaging, since Voltron and Positron, such as other rhodopsin-based GEVIs, are not compatible with two-photon imaging^7,14^. In neuron cultures, fluorescence responses to action potentials decayed at a similar rate for Positron and Voltron (Supplementary Fig. 9). Although currently Positron and Voltron perform interchangeably in vivo, we ran simulations to assess how future imaging of positive-going or negative-going GEVIs would be influenced by the density of labeled neurons and the sensitivity of the indicator, which our current experiments could not address. We found that for more densely labeled samples with higher background and for higher sensitivity indicators, positive-going GEVIs significantly outperform equivalent negative-going indicators (Supplementary Fig. 10). For example, GEVIs with a 5× larger fluorescence response to a neuron action potential allow imaging in 4× more densely labeled tissue using a positive-going indicator relative to a negative-going indicator with the same spike detection error rate (Supplementary Fig. 10c, d). Moreover, at high labeling density, the more sensitive positive-going indicator produces a 4× higher spike detection fidelity than its negative-going counterpart in our simulations (Supplementary Fig. 10f).

### A general engineering method for positive-going eFRET GEVIs

**Fig. 3:**
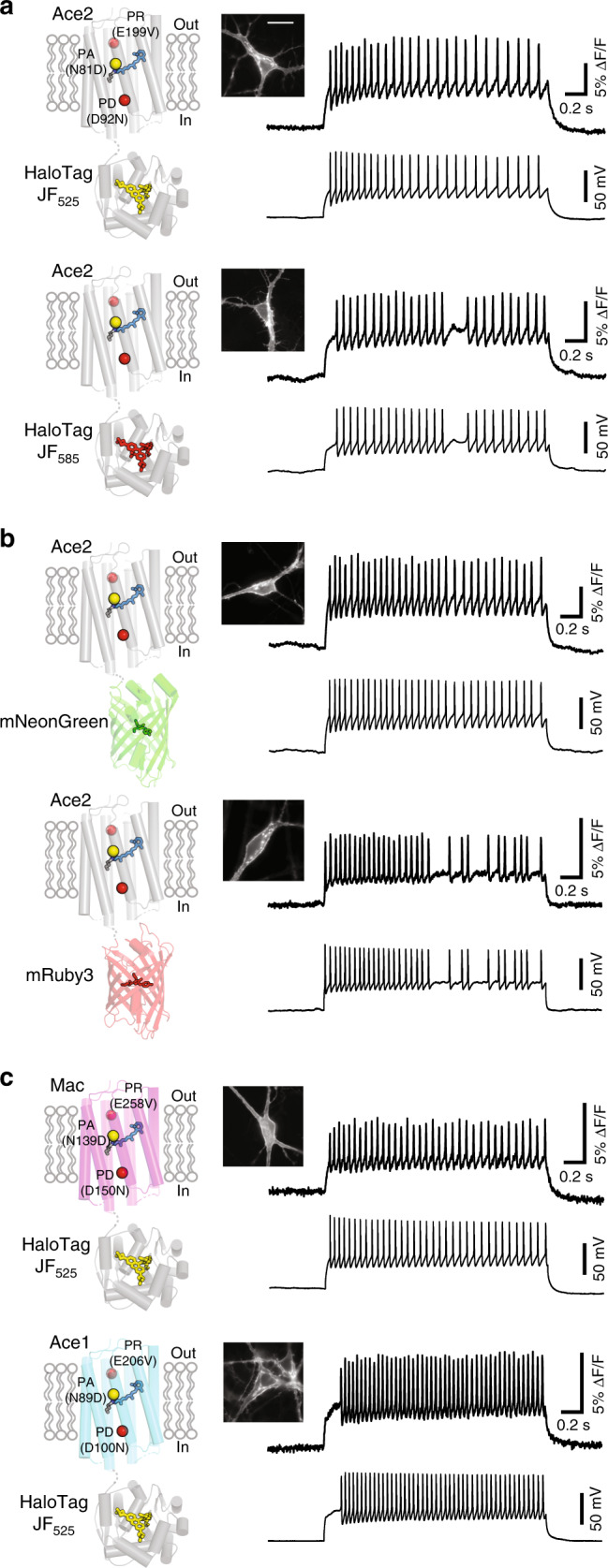
Generality of the approach to engineer positive-going eFRET GEVIs. Each panel shows a schematic of the voltage indicator construct (left), a fluorescence image of a neuron in culture expressing this voltage indicator (center), and simultaneous recording of fluorescence (right top) and membrane potential (right bottom) in response to current injection. Fluorescence image scale bar = 20 μm. Simultaneous voltage and fluorescence traces are representative of *N* ≥ 3 cells. **a** Positron labeled with two different colors of fluorescent dye, JF_525_ (top) and JF_585_ (bottom). **b** Ace2 rhodopsin bearing the signal-inverting mutations described and fused to the different color FP domains mNeonGreen (top) and mRuby3 (bottom). **c** Different rhodopsin domains (Mac, top and Ace1, bottom) bearing mutations analogous to the described signal-inverting mutations described, fused to HaloTag, and labeled with JF_525_.

To demonstrate the generality of our approach to generate eFRET GEVIs with positive-going fluorescence response, we explored different reporter fluorophores and rhodopsin domains. Positron showed sensitive fluorescence response when labeled with a yellow dye (JF_525_) or a red dye (JF_585_) (Fig. 3a). We also showed that the HaloTag could be exchanged for either green or red FP domains (Fig. 3b). We created positive-going versions of the Ace2N-mNeon^12^ and VARNAM^22^ eFRET GEVIs capable of sensitively reporting action potentials in neurons with positive-going signals (Fig. 3b and Supplementary Figs. 11 and 12). Next, we substituted the rhodopsin domain with Ace1, Mac, and Arch rhodopsins bearing mutations analogous to the three mutations we introduced to create Positron (Fig. 3c and Supplementary Figs. 13–18). Despite only low amino acid sequence identity (27–54%) between these rhodopsins (Supplementary Fig. 16), each of these permutations resulted in a GEVI with positive-going fluorescence changes capable of following action potentials when we patched neurons in culture and injected current (Fig. 3c and Supplementary Fig. 18). Importantly, all three rhodopsins bearing mutations analogous to Positron showed no steady-state photocurrent (Supplementary Figs. 17 and 18).

## Discussion

Positron is the first eFRET GEVI to run in reverse, having lower fluorescence at resting membrane potentials, positive-going signals in response to action potentials, and sensitivity equal to that of state-of-the-art eFRET GEVIs. We achieved this by rational mutation of the PD, PA, and PR sites within the characterized proton transport pathway of microbial rhodopsins and found that the effect of these substitutions can be generalized to other rhodopsins. This work provides further mechanistic insight into the class of rhodopsin eFRET GEVIs and puts forth a scaffold for future GEVI optimization.

## Methods

### Molecular biology

The genes for Ace2 and HaloTag were amplified from a Voltron plasmid^14^. The genes for Ace1, MacQ, QuasAr3, mNeonGreen, and mRuby3 were synthesized (Integrated DNA Technologies) with mammalian codon optimization^10–13,22^. Rhodopsin and fluorescence reporter genes were combined using overlap PCR. Cloning was done by restriction enzyme digest of plasmid backbones, PCR amplification of inserted genes, isothermal assembly, and followed by Sanger sequencing to verify DNA sequences. A soma localization tag^23,24^ (ST) was added using overlap PCR to make soma targeted versions of indicators (e.g. Positron-ST). For expression in primary neuron cultures, sensors were cloned into a pcDNA3.1-CAG plasmid (Invitrogen). For expression in zebrafish, Positron-ST was cloned into the pTol2-HuC vector (for pan-neuronal expression) and into the pT2-Tbait-UAS vector (for Gal4-dependent expression). The DNA and amino acid sequences of Positron, Ace1_Q89D_D100N_E206V-HaloTag, QuasAr3_Q95D_H106N_E214V-HaloTag, Mac_Q139D_D150N_E258V-HaloTag, Ace2_D92N_E199V-mNeonGreen, Ace2_D92N_E199V-mRuby3, are given in Supplementary Figs. 2 and 11–15. Plasmids and maps are available from Addgene.

### HEK 293 cell culture

Spiking HEK 293 cells^25^ that stably express NaV1.3 and Kir2.1 were used to test the fluorescence response of some Positron mutants using field stimulation. Cells were grown at 37 °C, 5% CO_2_, in Dulbecco’s modified Eagle medium supplemented with 10% fetal bovine serum, Geneticin (Invitrogen, 10131-027), Penicillin/Streptomycin (Invitrogen, 15140-122), and Puromycin (Invitrogen, A11138-03). All the transfections and assays were done in cells between passage 5 and 15. Plasmids were transfected using calcium phosphate. After transfection, spiking HEK cells were plated on glass-bottom 24-well plates (MaTeK) and cultured for 24 h before imaging. To label HaloTag-containing constructs, cells were incubated with 100 nM JF-dye HaloTag ligand for 30 min.

### Primary neuron cell culture

All procedures involving animals were conducted in accordance with protocols approved by the Howard Hughes Medical Institute Janelia Research Campus Institutional Animal Care and Use Committee and Institutional Biosafety Committee. Hippocampal neurons extracted from P0 to 1 Sprague–Dawley rat pups were transfected with pcDNA3.1-CAG plasmids of the various indicators by electroporation (Lonza, P3 Primary Cell 4D-Nucleofector X kit) according to the manufacturer’s instruction. After transfection, hippocampal neurons were plated onto 24-well glass-bottom plates (MaTek) or 35 mm glass-bottom dishes (MaTek) coated with poly-d-lysine (Sigma). Neurons were cultured for 8–12 days in NbActiv4 medium (BrainBits). To label neurons expressing Positron, Voltron, Ace1_Q89D_D100N_E206V-HaloTag, QuasAr3_Q95D_H106N_E214V-HaloTag, and Mac_Q139D_D150N_E258V-HaloTag, cultures were incubated with 100 nM JF HaloTag ligand for 20–30 mins.

### Microscopy

Cell cultures were illuminated with a SPECTRA X light engine (Lumencore) and observed through a ×40 oil objective (NA = 1.3, Nikon) on an inverted Nikon Eclipse Ti2 microscope. Excitation and emission light were passed through a FITC filter set (475/50 nm (excitation), 540/50 nm (emission), and a 506LP dichroic mirror (FITC-5050A-000; Semrock)) to image Ace2_D92N_E199V-mNeonGreen, a custom filter set (510/25 nm (excitation), 545/40 nm (emission), and a 525LP dichroic mirror (Chroma)) to image Voltron, Positron, Ace1_Q89D_D100N_E206V-HaloTag, and Mac_Q139D_D150N_E258V-HaloTag labeled with JF_525_, and a quad bandpass filter (set number: 89000, Chroma) with the appropriate color band from the SPECTRA X light source to image QuasAr3_Q95D_H106N_E214V-HaloTag labeled with JF_549_, Positron labeled with JF_585_, and Ace2_D92N_E199V-mRuby3. Fluorescence was collected onto a scientific CMOS camera (ORCA-Flash 4.0, Hamamatsu) and acquired using HCImage Live (Hamamatsu). For time-lapse imaging, images were acquired at 400–3200 Hz depending on the experiment. Imaging buffer (containing the following (in mM): 145 NaCl, 2.5 KCl, 10 glucose, 10 HEPES pH 7.4, 2 CaCl_2_, 1 MgCl_2_) was used in all cell culture experiments.

To process fluorescence images, a region of interest (ROI) was manually drawn around a neuron cell body in imageJ ﻿and the fluorescence was measured by averaging all pixels within the cell body. The raw fluorescence trace (F) is the mean intensity over the ROI in time. *F* is then fit with an exponential curve to account for bleaching. We calculated Δ*F*/*F* as *F* − *F*_0_/*F*_0_ with *﻿F*_0_ being the fluorescence baseline averaged over one second prior to stimulation.

To compare brightness of Positron and Voltron at resting membrane potential, we fused mTagBFP2^26^ to the C terminus of Positron and Voltron, to make pCAG-Positron-mTagBFP2 and pCAG-Voltron-mTagBFP2 plasmids. Fluorescence images were acquired from ten different wells across three independent transfections for each construct in primary hippocampal neurons. To label Voltron-mTagBFP2- and Positron-mTagBFP2-expressing neurons, cultures were incubated with 100 nM JF_525_ HaloTag ligand for 25 min, then washed twice with imaging buffer, incubating the cells for 5 min during the second wash. An EBFP2 filter set (405/20 nm (excitation), 460/50 nm (emission), and a 425LP dichroic mirror (49021; Chroma)) was used to image the TagBFP2 channel, and a custom filter set (510/25 nm (excitation), 545/40 nm (emission), and a 525LP dichroic mirror (Chroma)) was used to image the JF_525_ channel in Voltron-mTagBFP2 and Positron-mTagBF2 constructs. The ratio of the TagBFP2 channel and JF_525_ channel was calculated using ImageJ software.

To compare the photostability of Positron and Voltron at resting membrane potential, we used the soma localized versions, labeled with JF_525_ HaloTag ligand. Fluorescence images (100 ms exposure) were taken using a ×40 objective every second for 5 min of neuron cultures exposed to continuous 18 mW mm^−2^ light illumination from a light-emitting diode (LED) (Spectra X, Lumencore). After background subtraction, the average intensity per frame was calculated and plotted vs. time.

### Field stimulation in spiking HEK cells or neuron culture

A stimulus isolator (A385, World Precision Instruments) with platinum wires was used to deliver field stimuli (50 V, 1 ms) to elicit HEK cell spiking or action potentials in cultured neurons^27^. The stimulation was controlled using Wavesurfer and timing was synchronized with fluorescence acquisition using Wavesurfer and a National Instruments PCIe-6353 board.

To measure change of Positron and Voltron fluorescence response to action potentials over time, we labeled neurons expressing these indicators and labeled with JF_525_ HaloTag ligand and imaged with a ×40 objective at 400 Hz at 60 mW mm^−^^2^. We applied field electrode stimulations to induce a single action potential every 3 s. Individual neurons were manually segmented by drawing ROIs around the cell body. Change in fluorescence was calculated for each action potential of the trace as described above.

### Electrophysiology in primary neuron culture

Simultaneous whole-cell patch-clamp recordings along with fluorescence imaging were performed in cultured neurons. Current-clamp recordings were performed in imaging buffer: 145 NaCl, 2.5 KCl, 10 glucose, 10 HEPES pH 7.4, 2 CaCl_2_, 1 MgCl_2_, adjusted to 310 mOsm with sucrose at room temperature. For voltage-clamp recordings, 500 nM TTX was added to the imaging buffer to block sodium channels, and synaptic blockers (10 μM CNQX, 10 μM CPP, 10 μM GABAZINE, and 1 mM MCPG) were added to block ionotropic glutamate, GABA, and metabotropic glutamate receptors^27^.

Patch pipettes were fabricated using a P-97 puller (Sutter Instruments) to a tip resistance of 4–6 MΩ. For current-clamp recordings, pipettes were filled with the following (in mM): 130 potassium methanesulfonate, 10 HEPES, 5 NaCl, 1 MgCl_2_, 1 Mg-ATP, 0.4 Na-GTP, 14 Tris-phosphocreatine, adjusted to pH 7.3 with KOH, and adjusted to 300 mOsm with sucrose. For voltage-clamp recordings, a cesium-based internal solution was used ((in mM): 115 cesium methanesulfonate, 10 HEPES, 5 NaF, 10 EGTA, 15 CsCl, 3.5 Mg-ATP, 3 QX-314, adjusted to pH 7.3 with CsOH, and adjusted to 300 mOsm with sucrose).

Whole-cell recordings were made using an EPC800 amplifier (HEKA), filtered at 10 kHz with the internal Bessel filter, and digitized using a National Instruments PCIe-6353 acquisition board. Pipettes were positioned with a MPC200 manipulator (Sutter Instruments). Wavesurfer software was used to generate current injection waveforms, to record voltage and current traces, and to control the camera and light source. Neurons with access resistance >25 MΩ were discarded. For current-clamp recordings to generate action potentials, 20–200 pA for 1–2 s was injected and voltage was monitored.

For voltage-clamp experiments used to generate fluorescence-voltage curves, voltage steps (from −110 mV to +50 mV in 20 mV increments for 1 s) were applied to cells held at −70 mV. Fluorescence images were acquired at 400 Hz using the same microscope described in the “Microscopy” section above. For determining response speed of indicators, fluorescence images were acquired at 3200 Hz in response to a 100 mV potential step delivered to voltage-clamped neurons (from −70 mV to +30 mV). Traces were fit to a double exponential function using MATLAB. All recordings were done at room temperature.

### Photocurrent measurements

Photocurrents were recorded at room temperature in voltage-clamp mode with a holding potential of −70 mV in response to 1 s light pulses. Photocurrents were recorded using an EPC800 amplifier (HEKA), filtered at 10 kHz with an internal Bessel filter, and digitized using a National Instruments PCIe-6353 acquisition board at 20 kHz controlled using WaveSurfer. Light was delivered to the clamped neurons using the same microscope described above. Irradiance at the imaging plane was set to 70 mW mm^−2^ determined with a microscope slide power sensor (S170C, Thorlabs).

### Imaging in zebrafish

In vivo wide-field voltage imaging in zebrafish was performed. Briefly, the Positron-ST indicator was transiently expressed by the injection of pT2-Tbait-UAS-Positron-ST (25 ng μl^−1^ DNA with 25 ng μl^−1^ Tol2 transposase mRNA in E3 medium) in Tg(elavl3:Gal4-VP16) at 1–2 cell stage. JF_525_ was loaded to the injected fish at three day post-fertilization (dpf) by incubation in 3 μM JF_525_ system water solution for 2 h. The fish with sparsely labeled forebrain neurons were paralyzed by a-bungarotoxin (1 mg ml^−1^) and mounted in low-melting point agarose. Spontaneous activity of forebrain neurons was imaged using a custom wide-field microscope equipped with a ×60 1.0 NA water immersion objective lens (MRD07620, Nikon), a LED light source (CBT-90-W, Luminous) and a TRITC filter set (TRITC-B-000, Semrock). The images were acquired with sCMOS camera (pco.edge 4.2, PCO) at 400 Hz for 1–2 min. Irradiance at the imaging plane was 28 mW mm^−2^ (S170C, Thorlabs).

To compare the performance of the Positron-ST indicator and the Voltron-ST indicator to detect spiking activity, we focused on OSNs that showed tonic spontaneous spiking activity similar to those reported in other species^28,29^. JF_552_ HaloTag ligand^30^ was loaded using the same protocol described above and the fish with sparsely labeled OSNs were selected for imaging in order to minimize the contamination of signals from nearby cells. Using the same imaging protocol described above, fluorescent signal was recorded for 5 min. Raw fluorescent time course of individual OSNs was first extracted from manually drawn ROI and then the bleaching time constant of the dye for each indicator was calculated by fitting an exponential decay function to the raw time course. To examine percent signal change of transient spike-like signal, the baseline fluorescence signal corresponding to the resting membrane potential was estimated by the bottom 10th percentile of a 1 s moving time window (or the top 10th percentile signal in case of Voltron-ST) and used to calculate percent signal change for spike-like signal (Δ*F*/*F*_0_). Then the spike-like events were identified with the following procedure. First, Δ*F*/*F*_0_ was *z*-transformed and then maximal overlap discrete wavelet transform was performed using sym4 wavelet down to the sixth level. Then a frequency-localized version of the signal was reconstructed using the wavelet coefficients from the level 3 to 6 to maximize the energy of spike-like signal and then a threshold was applied its power to detect spike-like event. This procedure allowed us to detect clear spike-like signal independent of its percent signal change for both indicators with the exact same procedure and threshold. We restricted our analysis to the cells that showed tonic firing throughout the 5-min imaging session and examined amplitude (Δ*F*/*F*_0_) and SNR (*z*-score).

### Simulations of positive- and negative- going GEVIs

To simulate performance of positive- vs. negative-going indicators, we started with a real recording from superficial mouse cortex and added simulated neurons as in our previous work^14^. In these simulations, we used a real recording (40,000 frames) containing a single Voltron-labeled neuron as a positive control. We synthesized spatial footprints for each neuron. We then simulated ‘voltage traces’ for each neuron (Supplementary Fig. 10b), containing a mean of 100 spikes per recording, and converted these to either positive and negative-going indicator traces ranging between *F*_min_ and *F*_max_. For positive-going traces, *F*_min_ corresponds to the fluorescence at the resting membrane potential and *F*_max_ to the mean spike height. For negative-going traces, *F*_min_ corresponds to the mean spike height and *F*_max_ to the fluorescence at the resting membrane potential.

Indicator traces were multiplied by the empirical bleaching curve estimated from the neuron in the video and scaled to a comparable brightness. These model neuron recordings were summed across neurons and Gaussian noise proportional to signal was added to each pixel. This sampled simulated recording was then added to the real recording to produce the simulation instance for analysis.

We simulated imaging of different numbers of neurons with different indicator sensitivities. For each simulated neuron for which we estimated spikes, we included 5 additional ‘out of focus’ neurons with blurred spatial footprints and temporally shuffled spiking intended to model background sources in real recordings. For different indicator sensitivities (Δ*F*/*F*_min_), we held *F*_max_ fixed (to simulate a fixed maximum brightness of the dye ligand) and varied F_min_ to produce the appropriate Δ*F*/*F*_min_. Spikes were recovered using the Spike Pursuit algorithm^14^. We plot the error rate of spike inference, calculated as 1-IoU, where IoU is the intersection divided by the union of binary spike labels for the ground truth and inferred spikes at the simulated imaging rate 400 Hz, without binning. Code used in simulations is available from github.com/KasparP/Positron.

### Reporting summary

Further information on research design is available in the Nature Research Reporting Summary linked to this article.

## Supplementary information

Supplementary Information

Reporting Summary

## Data Availability

All DNA and protein sequences described have been deposited at Addgene (www.addgene.org) as follows: pCAG-Positron (plasmid# 129253), pCAG-Positron-ST (plasmid# 129254), pCAG-Ace1_Q89D_D100N_E206V-HaloTag (plasmid# 129255), pCAG-Ace1_Q89D_D100N_E206V-HaloTag-ST (plasmid# 129260), pCAG-Ace2_D92N_E199V-mNeon (plasmid# 129256), pCAG-Ace2_D92N_E199V-mNeon-ST (plasmid# 129261), pCAG-Ace2_D92N_E199V-mRuby3 (plasmid# 129257), pCAG-Ace2_D92N_E199V-mRuby3-ST (plasmid# 129262), pCAG-QuasAr3_Q95D_H106N_E214V-HaloTag (plasmid# 129258), pCAG-QuasAr3_Q95D_H106N_E214V-HaloTag-ST (plasmid# 129263), pCAG-Mac_Q139D_D150N_E258V-HaloTag (plasmid# 129259), pCAG-Mac_Q139D_D150N_E258V-HaloTag-ST (plasmid# 129264), pTol2-Huc-Positron-ST (plasmid# 129265), pT2-Tbait-UAS-Positron-ST (plasmid# 129266), and pAAV-hsyn-flex-Positron-ST (plasmid# 129267). Source data from experiments in this study are available from the authors upon reasonable request.

## References

[1] Xu Y, Zou P, Cohen AE (2017). Voltage imaging with genetically encoded indicators. Curr. Opin. Chem. Biol..

[2] Lin MZ, Schnitzer MJ (2016). Genetically encoded indicators of neuronal activity. Nat. Neurosci..

[3] Jin L (2012). Single action potentials and subthreshold electrical events imaged in neurons with a fluorescent protein voltage probe. Neuron.

[4] Abdelfattah AS (2016). A bright and fast red fluorescent protein voltage indicator that reports neuronal activity in organotypic brain slices. J. Neurosci..

[5] St-Pierre F (2014). High-fidelity optical reporting of neuronal electrical activity with an ultrafast fluorescent voltage sensor. Nat. Neurosci..

[6] Yang HHH (2016). Subcellular imaging of voltage and calcium signals reveals neural processing in vivo. Cell.

[7] Chamberland S (2017). Fast two-photon imaging of subcellular voltage dynamics in neuronal tissue with genetically encoded indicators. Elife.

[8] Kralj JM, Douglass AD, Hochbaum DR, Maclaurin D, Cohen AE (2011). Optical recording of action potentials in mammalian neurons using a microbial rhodopsin. Nat. Methods.

[9] Hochbaum DR (2014). All-optical electrophysiology in mammalian neurons using engineered microbial rhodopsins. Nat. Methods.

[10] Adam Y (2019). Voltage imaging and optogenetics reveal behaviour-dependent changes in hippocampal dynamics. Nature.

[11] Zou P (2014). Bright and fast multicoloured voltage reporters via electrochromic FRET. Nat. Commun..

[12] Gong Y (2015). High-speed recording of neural spikes in awake mice and flies with a fluorescent voltage sensor. Science.

[13] Gong Y, Wagner MJ, Zhong Li J, Schnitzer MJ (2014). Imaging neural spiking in brain tissue using FRET-opsin protein voltage sensors. Nat. Commun..

[14] Abdelfattah AS (2019). Bright and photostable chemigenetic indicators for extended in vivo voltage imaging. Science.

[15] Chow BY (2010). High-performance genetically targetable optical neural silencing by light-driven proton pumps. Nature.

[16] Platisa J, Vasan G, Yang A, Pieribone VA (2017). Directed evolution of key residues in fluorescent protein inverses the polarity of voltage sensitivity in the genetically encoded indicator ArcLight. ACS Chem. Neurosci..

[17] Wada T (2011). Crystal structure of the eukaryotic light-driven proton-pumping rhodopsin, Acetabularia rhodopsin II, from marine alga. J. Mol. Biol..

[18] Grimm JB (2015). A general method to improve fluorophores for live-cell and single-molecule microscopy. Nat. Methods.

[19] Grimm JB (2017). A general method to fine-tune fluorophores for live-cell and in vivo imaging. Nat. Methods.

[20] Lanyi, J. K. Proton transfers in the bacteriorhodopsin photocycle. *Biochim. Biophys. Acta Bioenergetics* **1757**, 1012–1018 (2006). 10.1016/j.bbabio.2005.11.003 16376293

[21] Piatkevich KD (2018). A robotic multidimensional directed evolution approach approach applied to fluorescent voltage reporters. Nat. Chem. Biol..

[22] Kannan M (2018). Fast, in vivo voltage imaging using a red fluorescent indicator. Nat. Methods.

[23] Baker CA, Elyada YM, Parra A, Bolton MML (2016). Cellular resolution circuit mapping with temporal-focused excitation of soma-targeted channelrhodopsin. Elife.

[24] Lim ST, Antonucci DE, Scannevin RH, Trimmer JS (2000). A novel targeting signal for proximal clustering of the Kv2.1 K+channel in hippocampal neurons. Neuron.

[25] Park J (2013). Screening fluorescent voltage indicators with spontaneously spiking HEK cells. PLoS ONE.

[26] Subach OM, Cranfill PJ, Davidson MW, Verkhusha VV (2011). An enhanced monomeric blue fluorescent protein with the high chemical stability of the chromophore. PLoS ONE.

[27] Wardill TJ (2013). A neuron-based screening platform for optimizing genetically-encoded calcium indicators. PLoS ONE.

[28] Hallem EA, Ho MG, Carlson JR (2004). The molecular basis of odor coding in the Drosophila antenna. Cell.

[29] Connelly T, Savigner A, Ma M (2013). Spontaneous and sensory-evoked activity in mouse olfactory sensory neurons with defined odorant receptors. J. Neurophysiol..

[30] Zheng Q (2019). Rational design of fluorogenic and spontaneously blinking labels for super-resolution imaging. ACS Cent. Sci..

