# Supplementary material for: A general approach to engineer positive-going eFRET voltage indicators
Source: Nat Commun. 2020 Jul 10;11:3444. doi: 10.1038/s41467-020-17322-1 (PMC7351947; doi:10.1038/s41467-020-17322-1)
Supplement: Supplementary file 1 — Supplementary Information [file 41467_2020_17322_MOESM1_ESM.pdf]

Supplementary Materials for

A general approach to engineer positive-going eFRET voltage indicators

Ahmed S. Abdelfattah[1], Rosario Valenti[1], Jihong Zheng[1], Allan Wong[1], GENIE Project Team[1], Kaspar Podgorski[1], Minoru Koyama[1], Douglas S. Kim[1], Eric R. Schreiter[1]

[1]Janelia Research Campus, Howard Hughes Medical Institute, Ashburn, VA, USA

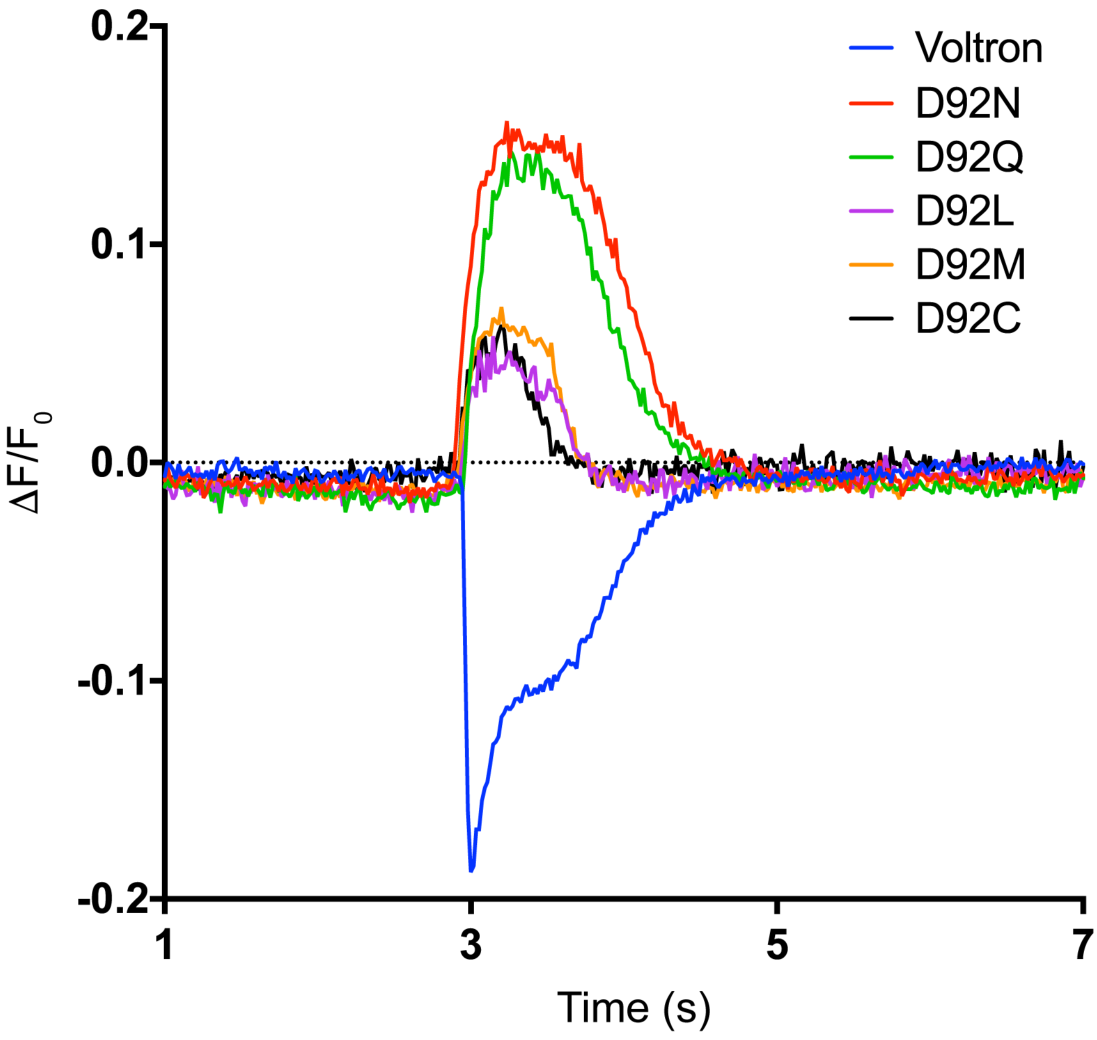

Supplementary Figure 1. Spiking HEK cell fluorescence traces for Voltron variants with mutations at position D92. Cells were subject to field stimulation at t = 3 seconds. Saturation mutagenesis at the proton donor position of Ace2 (D92) results in variants with a positive fluorescence change in response to membrane depolarization. All Voltron variants were labeled with JF_{525}. Fluorescence traces are representative of N = 4 wells of cells.

```
Ace2
atggctgacgtggaaaccgagaccggcatgattgcacagtggattgtctttgctattatggctgctgctgctattgcttttggagtggctgtgcactttc
 M  A  D  V  E  T  E  T  G  M  I  A  Q  W  I  V  F  A  I  M  A  A  A  A  I  A  F  G  V  A  V  H  F
Ace2
ggccttcagagctgaagagcgcatactatatcaacattgccatctgcactatcgccgctaccgcttactatgcaatggccgtgaactaccaggacctgac
R  P  S  E  L  K  S  A  Y  Y  I  N  I  A  I  C  T  I  A  A  T  A  Y  Y  A  M  A  V  N  Y  Q  D  L  T
Ace2
                                         PA                                  PD
aatgaatggtgaaaggcaggtggtctacgcaagatatattgactgggtgctgaccacaccactgctcctgctcaacctcatcgtcatgaccaagatgggc
  M  N  G  E  R  Q  V  V  Y  A  R  Y  I  D  W  V  L  T  T  P  L  L  L  L  N  L  I  V  M  T  K  M  G
Ace2
ggagtgatgatttcttgggtcatcggcgcagacattttcatgatcgtgtttggtattctgggcgccttcgaggatgaacacaagttcaaatgggtgtact
 G  V  M  I  S  W  V  I  G  A  D  I  F  M  I  V  F  G  I  L  G  A  F  E  D  E  H  K  F  K  W  V  Y
Ace2
ttatcgctggatgtgtgatgcaggcagtcctgacatacgggatgtataacgccacttggaaagacgatctgaagaaaagccccgagtaccatagctccta
F  I  A  G  C  V  M  Q  A  V  L  T  Y  G  M  Y  N  A  T  W  K  D  D  L  K  K  S  P  E  Y  H  S  S  Y
Ace2
                                                                                                  PR
tgtcagtctgctcgtcttcctgtcaatcctctgggtgttttatcctgtcgtgtgggctttcgggtctggtagtggcgtgctgtccgtcgacaatgtggcc
  V  S  L  L  V  F  L  S  I  L  W  V  F  Y  P  V  V  W  A  F  G  S  G  S  G  V  L  S  V  D  N  V  A
Ace2                                                                                        HaloTag
attctcatgggaatcctggatgtgctcgctaagccactgtttggaatggggtgcctcattgcccatgagactatcttcaagaagatgatcggtactggct
 I  L  M  G  I  L  D  V  L  A  K  P  L  F  G  M  G  C  L  I  A  H  E  T  I  F  K  K  M  I  G  T  G
HaloTag
ttccattcgaccccattatgtggaagtcctgggcgagcgcatgcactacgtcgatgttggtccgcgcgatggcacccctgtgctgttcctgcacggtaa
F  P  F  D  P  H  Y  V  E  V  L  G  E  R  M  H  Y  V  D  V  G  P  R  D  G  T  P  V  L  F  L  H  G  N
HaloTag
cccgacctcctcctacgtgtggcgcaacatcatcccgcatgttgcaccgacccatcgctgcattgctccagacctgatcggtatgggcaaatccgacaaa
  P  T  S  S  Y  V  W  R  N  I  I  P  H  V  A  P  T  H  R  C  I  A  P  D  L  I  G  M  G  K  S  D  K
HaloTag
ccagacctgggttatttcttcgacgaccacgtccgcttcatggatgccttcatcgaagccctgggtctggaagaggtcgtcctggtcattcacgactggg
 P  D  L  G  Y  F  F  D  D  H  V  R  F  M  D  A  F  I  E  A  L  G  L  E  E  V  V  L  V  I  H  D  W
HaloTag
gctccgctctgggtttccactgggccaagcgcaatccagagcgcgtcaaaggtattgcatttatggagttcatccgccctatcccgacctgggacgaatg
G  S  A  L  G  F  H  W  A  K  R  N  P  E  R  V  K  G  I  A  F  M  E  F  I  R  P  I  P  T  W  D  E  W
HaloTag
gccagaatttgcccgcgagaccttccaggccttccgcaccaccgacgtcggccgcaagctgatcatcgatcagaacgtttttatcgagggtacgctgccg
  P  E  F  A  R  E  T  F  Q  A  F  R  T  T  D  V  G  R  K  L  I  I  D  Q  N  V  F  I  E  G  T  L  P
HaloTag
atgggtgtcgtccgcccgctgactgaagtcgagatggaccattaccgcgagccgttcctgaatcctgttgaccgcgagccactgtggcgcttcccaaacg
 M  G  V  V  R  P  L  T  E  V  E  M  D  H  Y  R  E  P  F  L  N  P  V  D  R  E  P  L  W  R  F  P  N
HaloTag
agctgccaatcgccggtgagccagcgaacatcgtcgcgctggtcgaagaatacatggactggctgcaccagtcccctgtcccgaagctgctgttctgggg
E  L  P  I  A  G  E  P  A  N  I  V  A  L  V  E  E  Y  M  D  W  L  H  Q  S  P  V  P  K  L  L  F  W  G
HaloTag
caccccaggcgttctgatcccaccggccgaagccgctcgcctggccaaaagcctgcctaactgcaaggctgtggacatcggcccgggtctgaatctgctg
  T  P  G  V  L  I  P  P  A  E  A  A  R  L  A  K  S  L  P  N  C  K  A  V  D  I  G  P  G  L  N  L  L
HaloTag                                                                    Kir2.1 membrane ...fficking signal
caagaagacaacccggacctgatcggcagcgagatcgcgcgctggctgtcgacgctcgagatttccggcgagccaaccactaagagcaggatcaccagcg
  Q  E  D  N  P  D  L  I  G  S  E  I  A  R  W  L  S  T  L  E  I  S  G  E  P  T  T  K  S  R  I  T  S
Kir2.1 membrane trafficking signal       Kir2.1 ER export signal
agggcgagtacatcccccctggaccagatcgacatcaacgtgttctgctacgagaacgaggtgtaa
E  G  E  Y  I  P  L  D  Q  I  D  I  N  V  F  C  Y  E  N  E  V  .
```

Supplementary Figure 2. DNA and amino acid sequence of Positron with sequence features annotated. Note the three point mutations that are responsible for the positive fluorescence voltage slope annotated as PA (proton acceptor position), PD (proton donor position), and PR (proton release position).

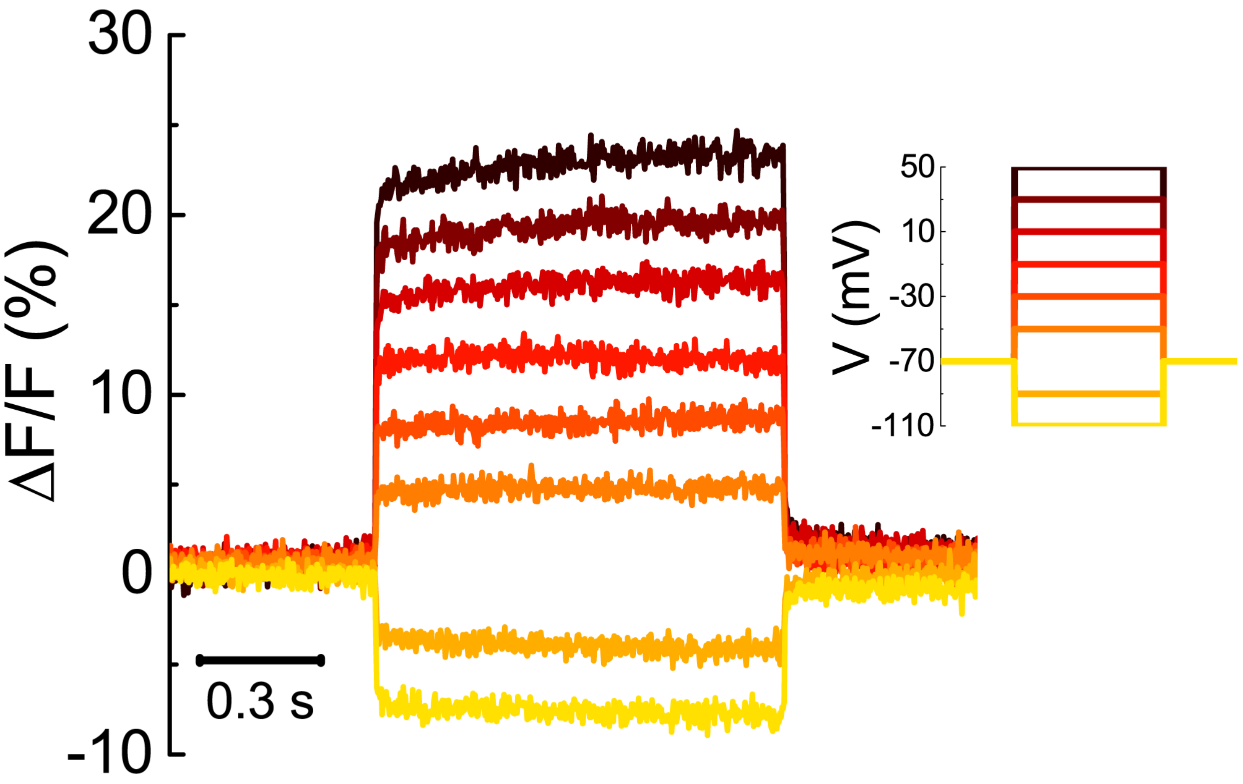

Supplementary Figure 3. Fluorescence traces of Positron labeled with JF_{525} in response to a series of voltage steps (from -110 mV to +50 mV in 20 mV increments). Image acquisition rate = 400 Hz. For complete fluorescence vs voltage plots of Positron, Voltron_N81D_D92N, and Voltron see Figure 1g. Representative of N = 5 cells.

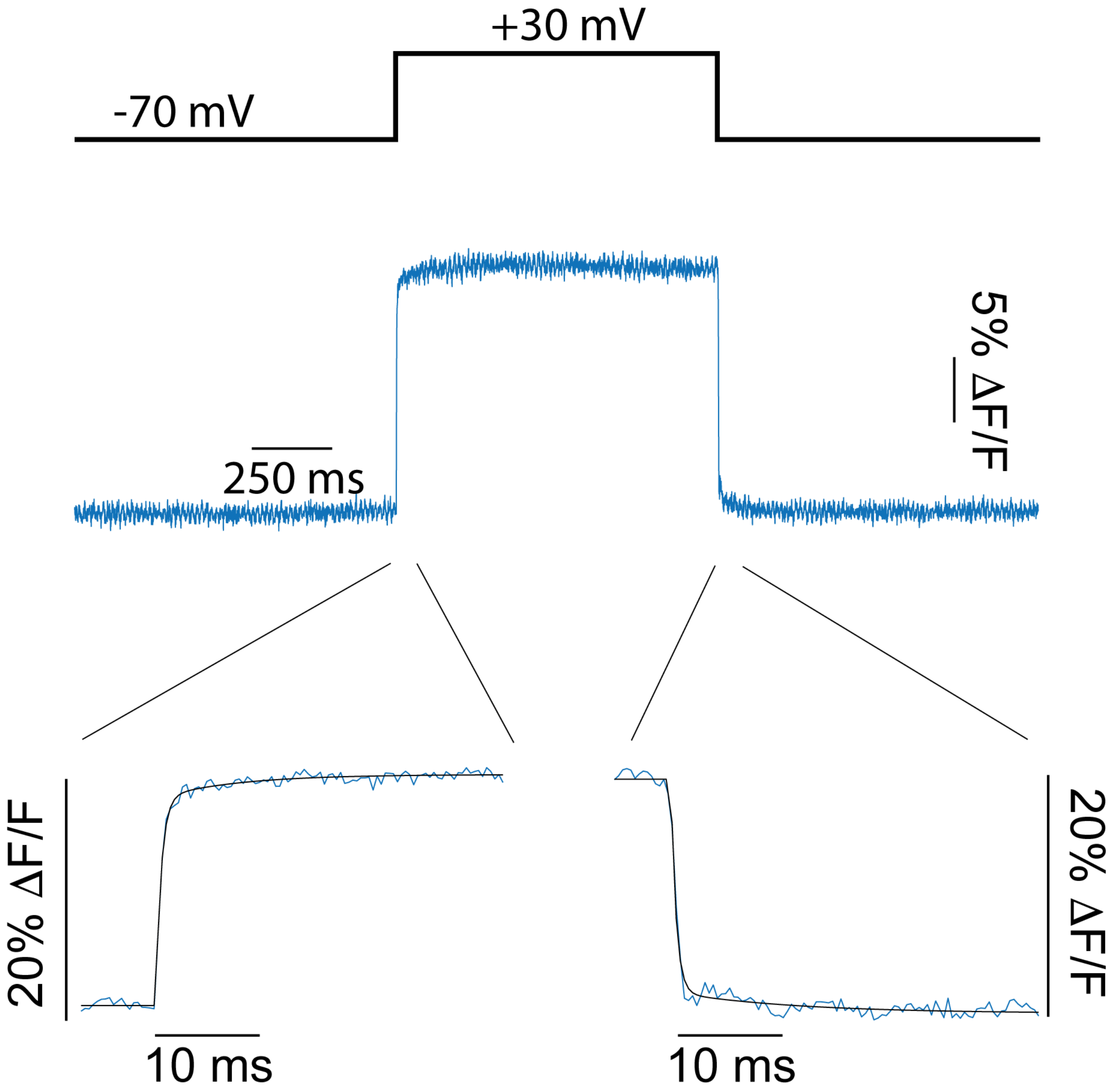

Supplementary Figure 4. Fluorescence response of Positron labeled with JF_{525} in response to a 100 mV potential step delivered to a voltage clamped neuron. Insets: Zoom in on Positron fluorescence response to membrane depolarization (from -70 mV to +30 mV), and repolarization (from +30 mV to -70 mV). Solid black line is fit of rise and decay kinetics to a double exponential function. Image acquisition rate 3.2 kHz. For full kinetics data, see Supplementary Table 1.

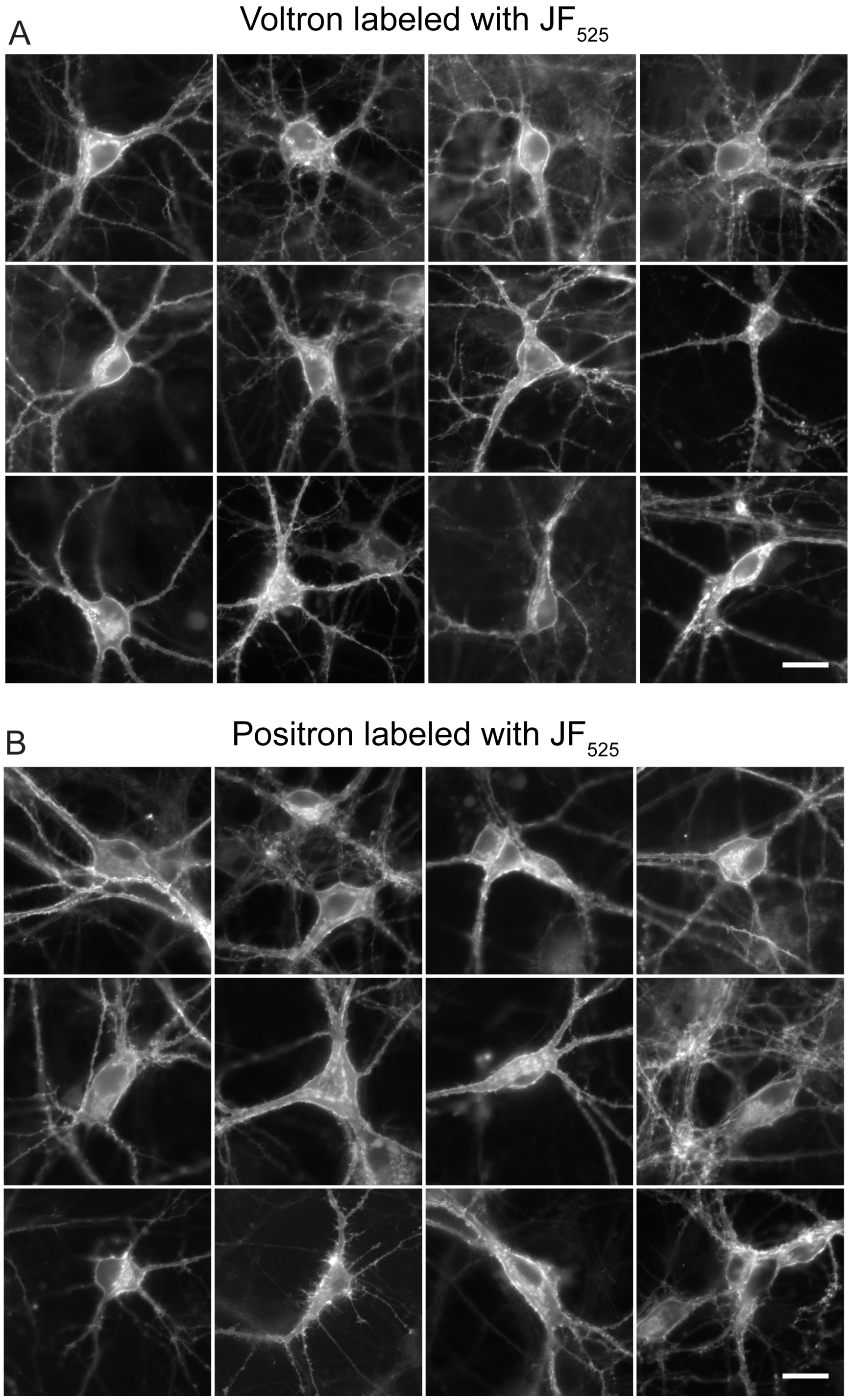

Supplementary Figure 5. Fluorescence images of primary neuron cultures expressing (A) Voltron and (B) Positron labeled with JF_{525}. Scale bar = 20µm. Fluorescence images were acquired from 9 different wells across three independent transfections for each construct in primary neuron cultures.

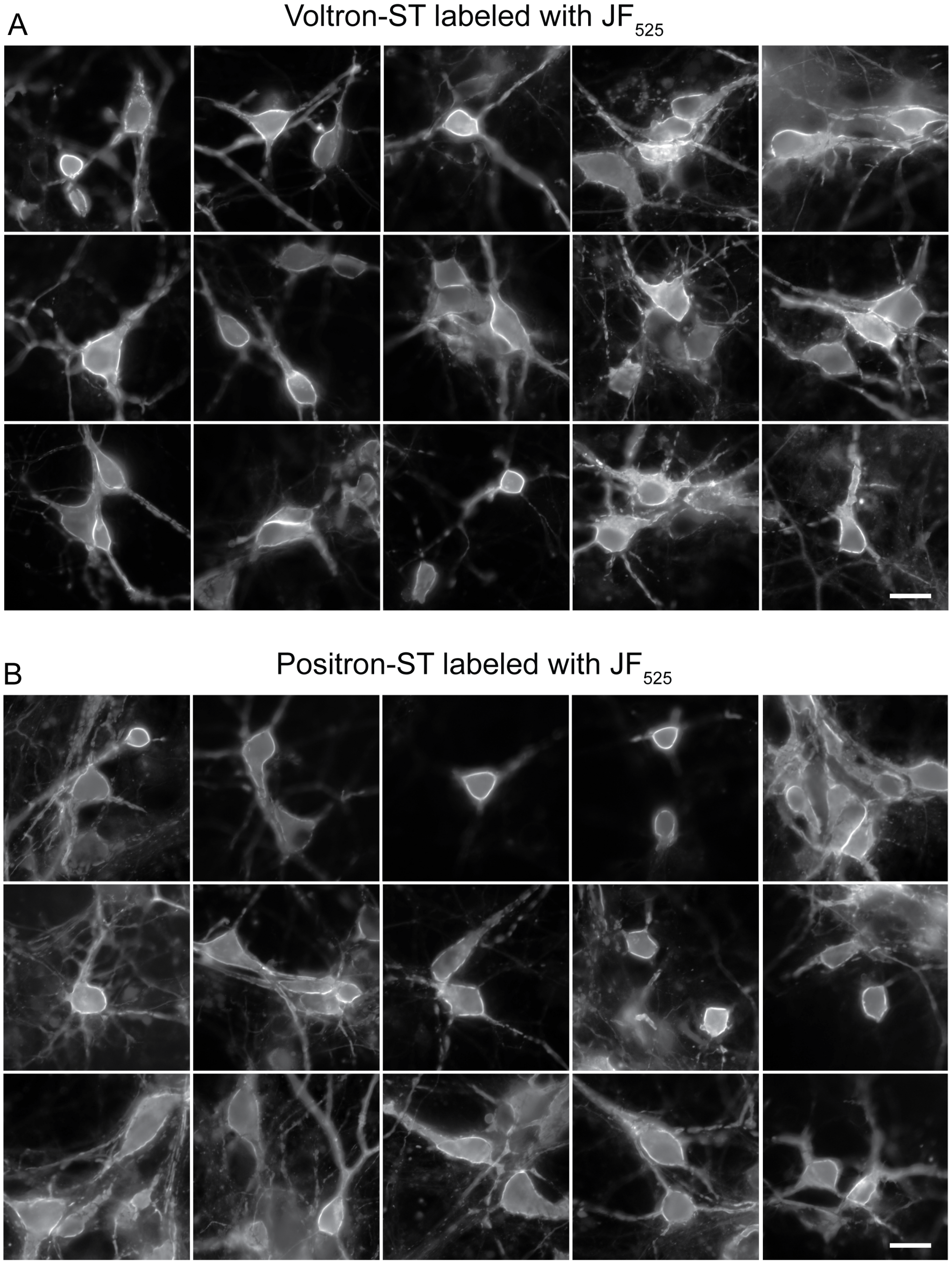

Supplementary Figure 6. Fluorescence images of primary neuron cultures expressing (A) Voltron-ST and (B) Positron-ST labeled with JF_{525}. Scale bar = 20µm. Fluorescence images were acquired from 9 different wells across three independent transfections for each construct in primary neuron cultures.

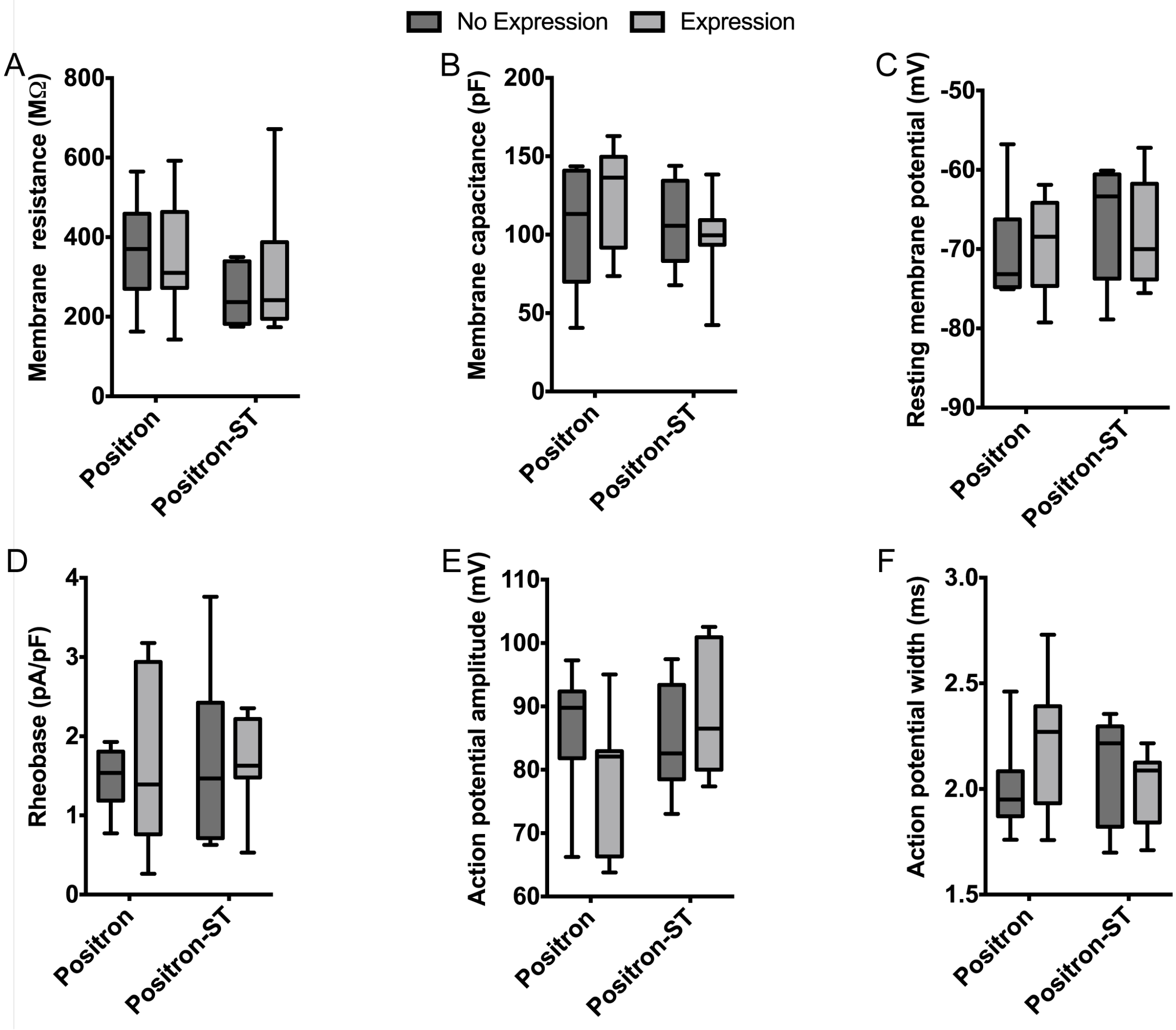

Supplementary Figure 7. Neuronal electrophysiology parameters of Positron and Voltron-ST expressing neurons labeled with JF_{525}. Box and whisker plots of (A) membrane resistance, (B) membrane capacitance, (C) resting membrane potential, (D) rheobase, (E) action potential amplitude, and (F) action potential width from cultured rat hippocampal neurons. Box and whisker plots: box = interquartile range (25–75 percentile), center = median value, whiskers = 5–95 percentile. Expressing cells were compared to non-expressing cells from the same set of dishes. N = 7 neurons for Positron untransfected cells, Positron expressing cells, and Positron-ST expressing cells. N = 8 neurons for Positron-ST untransfected cells.

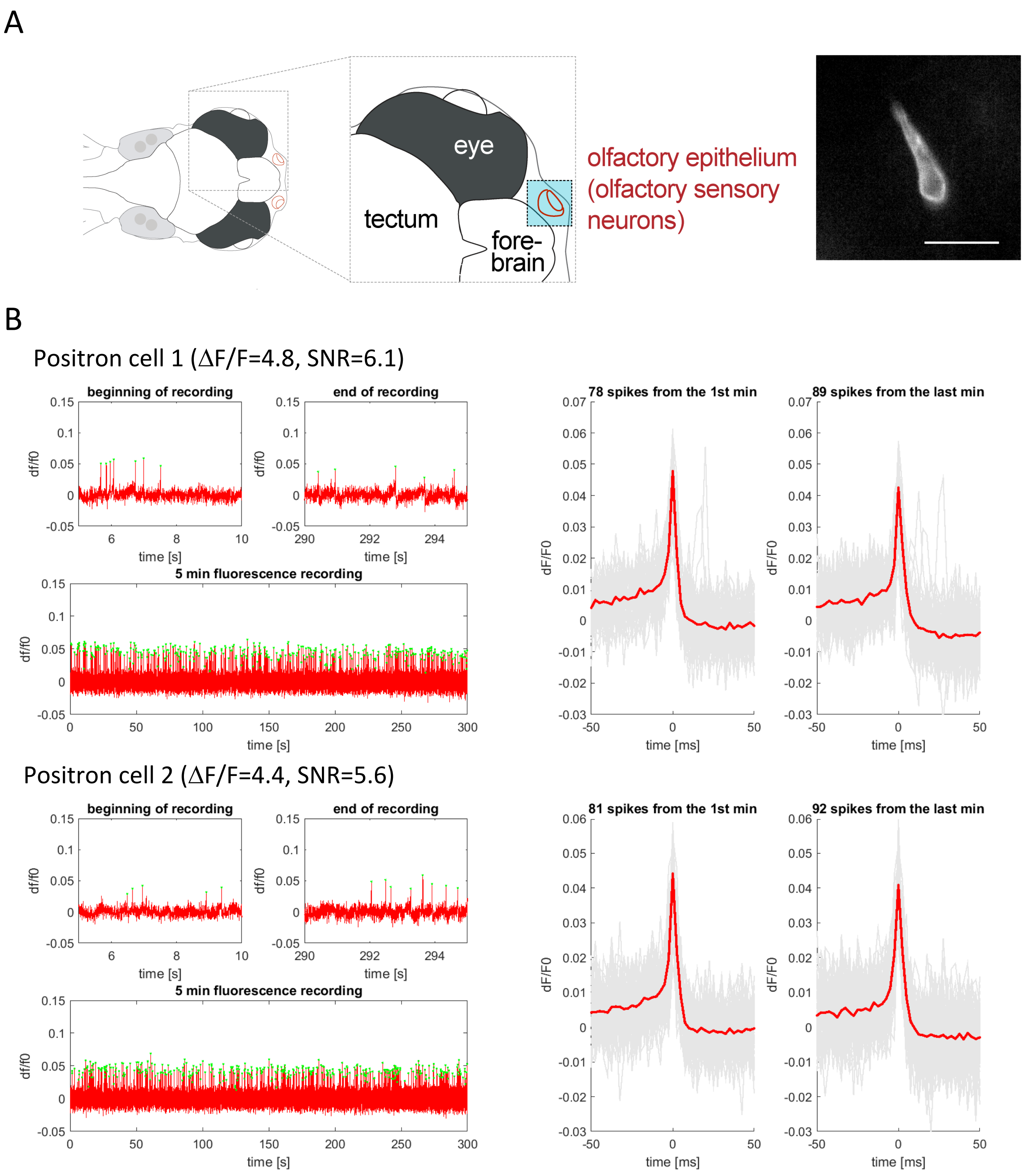
A
eye
tectum
fore-
brain
olfactory epithelium
(olfactory sensory
neurons)
B
Positron cell 1 (ΔF/F=4.8, SNR=6.1)
beginning of recording
end of recording
df/f0
time [s]
5 min fluorescence recording
78 spikes from the 1st min
89 spikes from the last min
dF/F0
time [ms]
Positron cell 2 (ΔF/F=4.4, SNR=5.6)
beginning of recording
end of recording
5 min fluorescence recording
81 spikes from the 1st min
92 spikes from the last min

Positron cell 3 (ΔF/F=5.3, SNR=4.8)

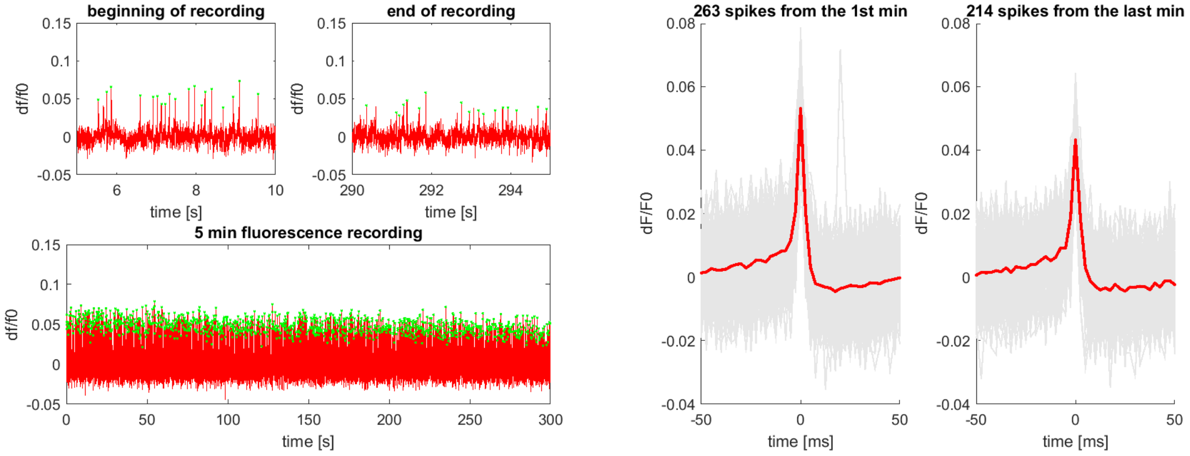

Positron cell 4 (ΔF/F=9.5, SNR=4.1)

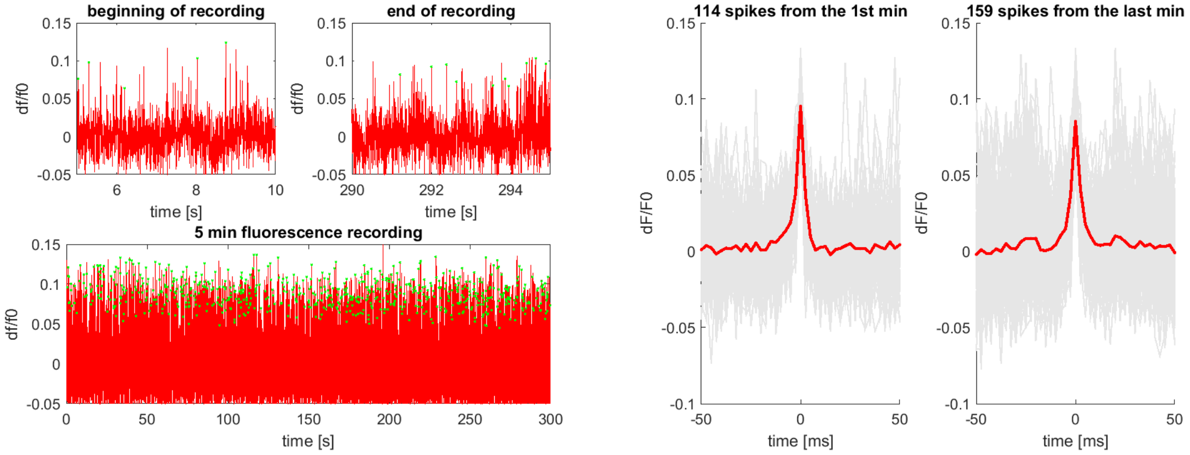

Positron cell 5 (ΔF/F=3.7, SNR=3.7)

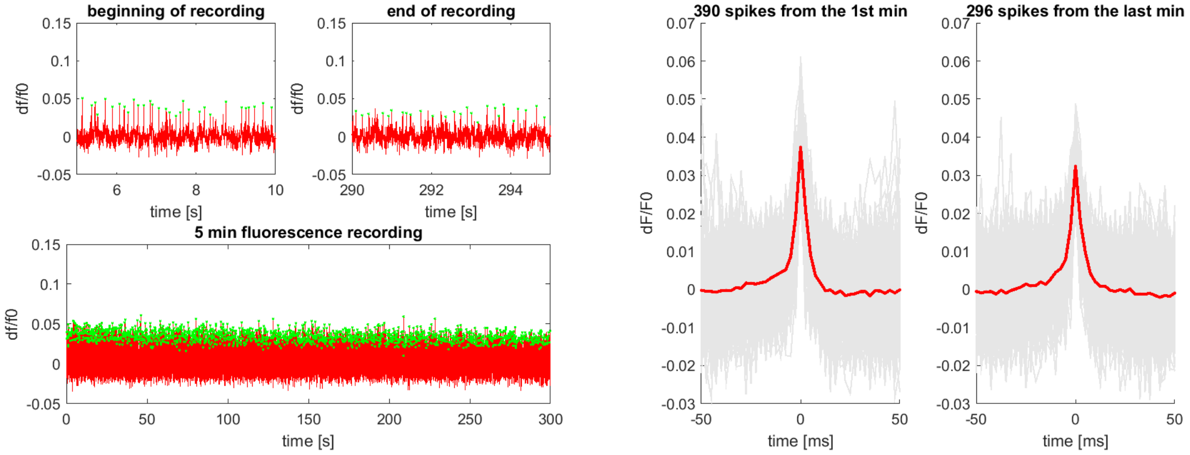

Positron cell 6 (ΔF/F=4.6, SNR=4.5)

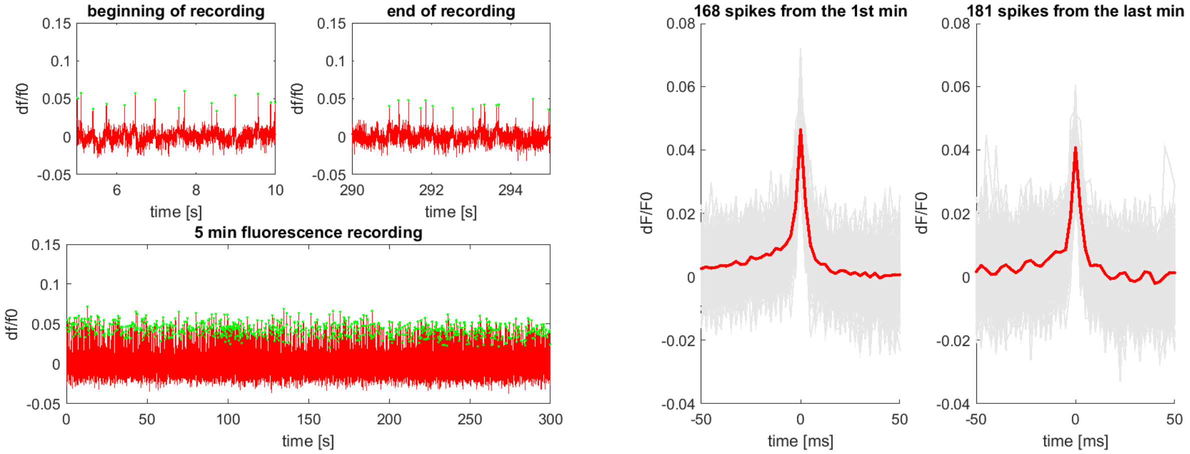

Positron cell 7 (ΔF/F=3.7, SNR=5.0)

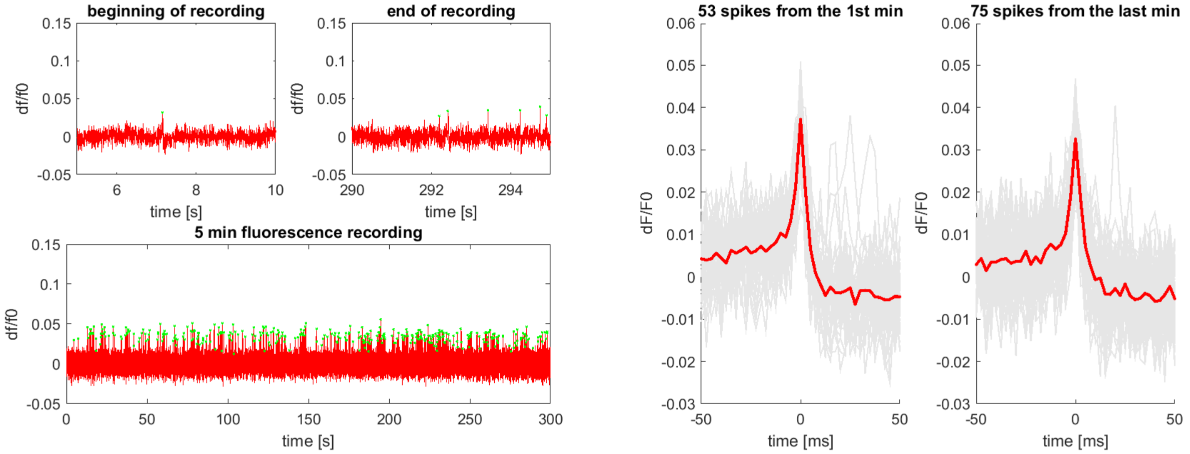

Positron cell 8 (ΔF/F=4.3, SNR=4.1)

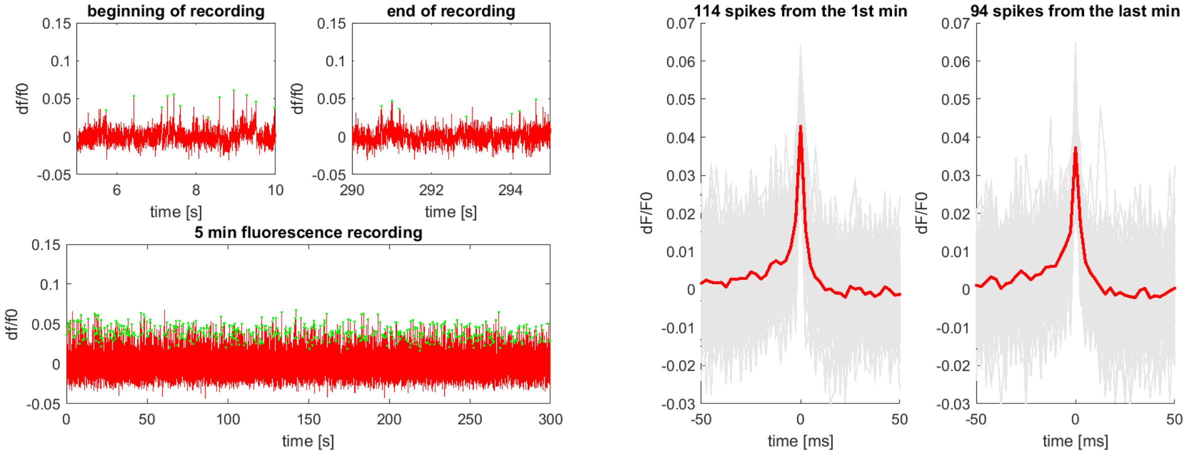

Positron cell 9 ($\Delta F/F=4.9$, SNR=3.7)

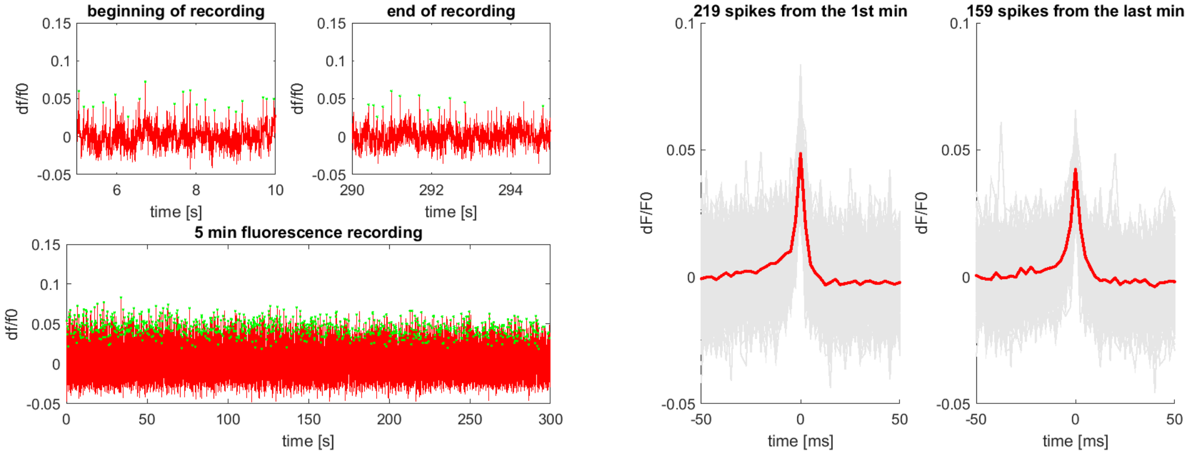

Positron cell 10 ($\Delta F/F=4.9$, SNR=2.7)

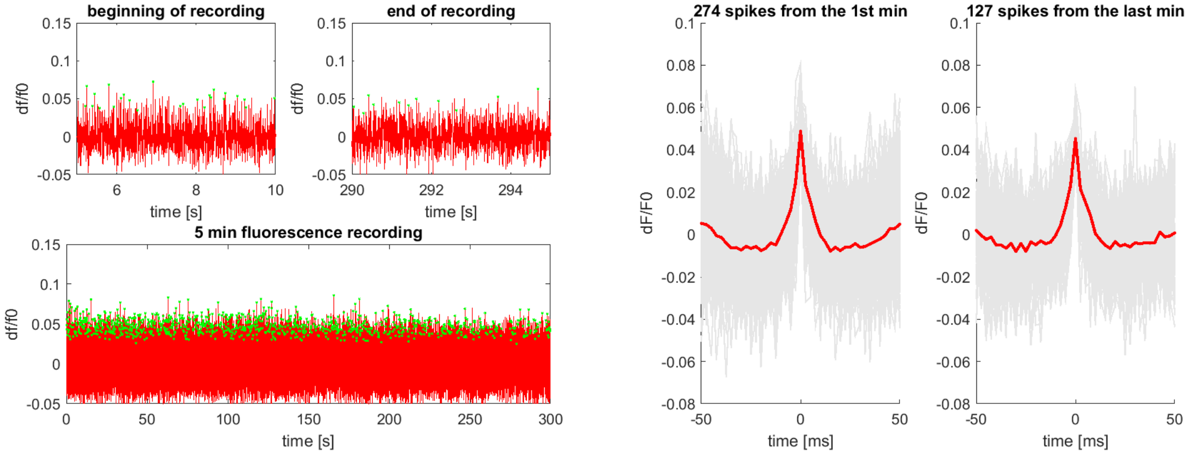

Voltron cell 1 ($\Delta F/F=4.7$, SNR=4.4)

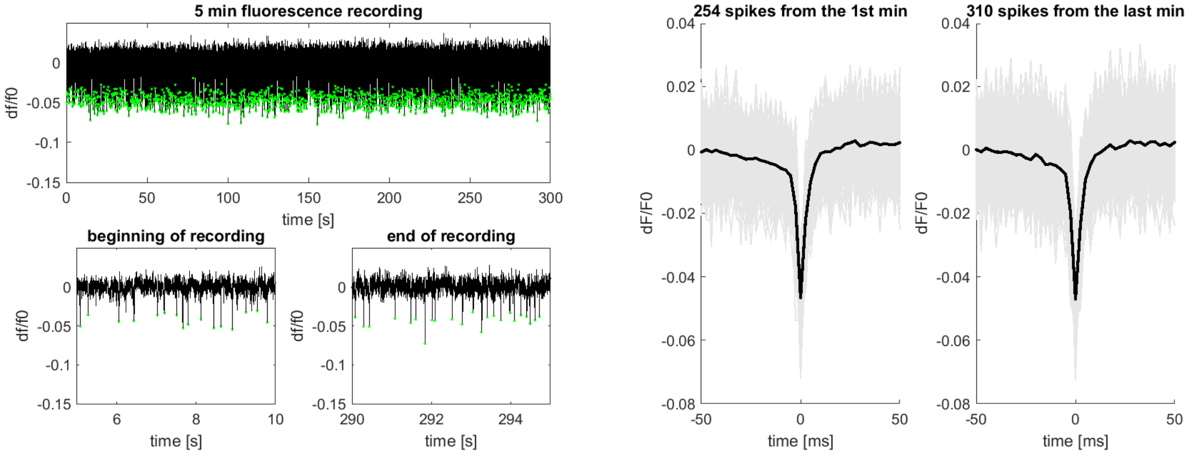

Voltron cell 2 (ΔF/F=6.0, SNR=5.2)

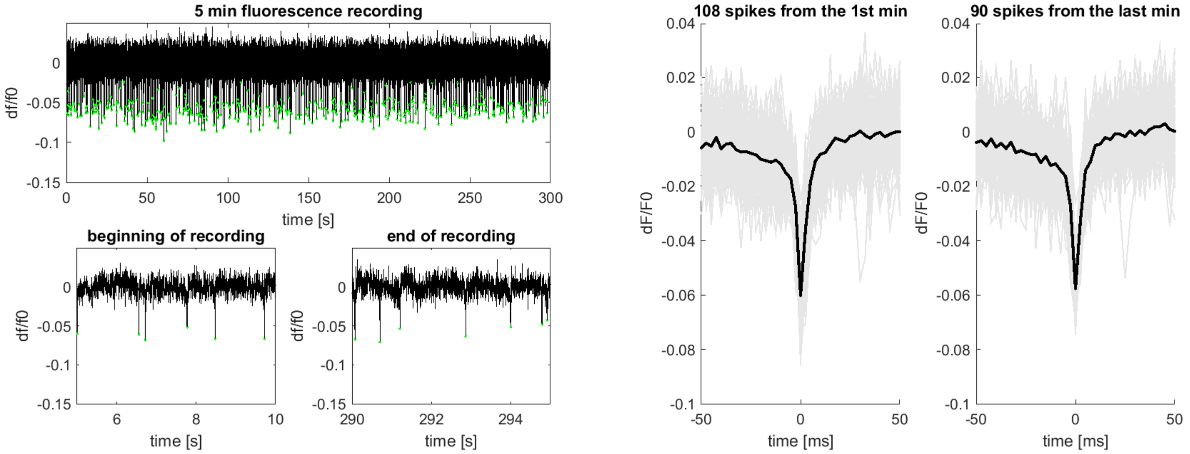

Voltron cell 3 (ΔF/F=8.8, SNR=3.2)

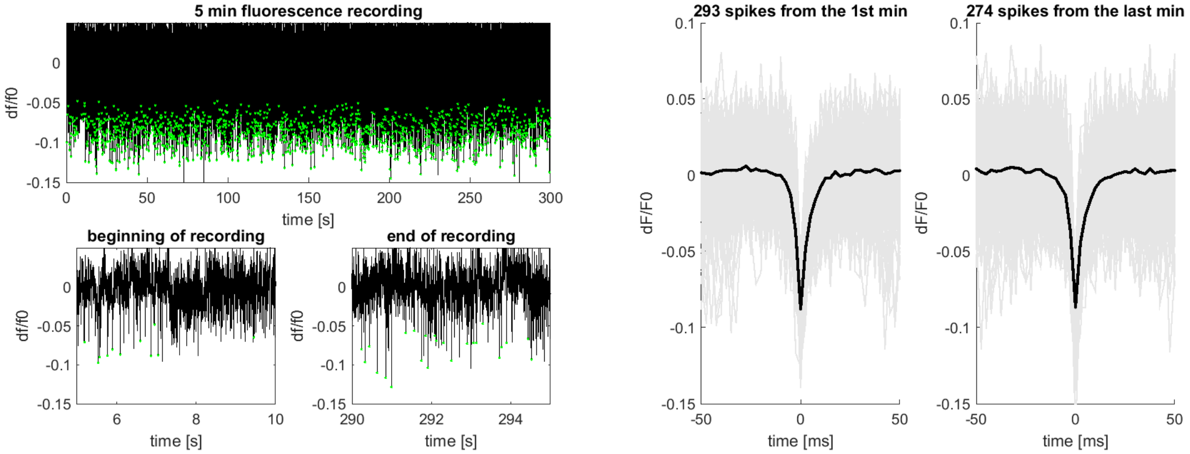

Voltron cell 4 (ΔF/F=6.1, SNR=3.1)

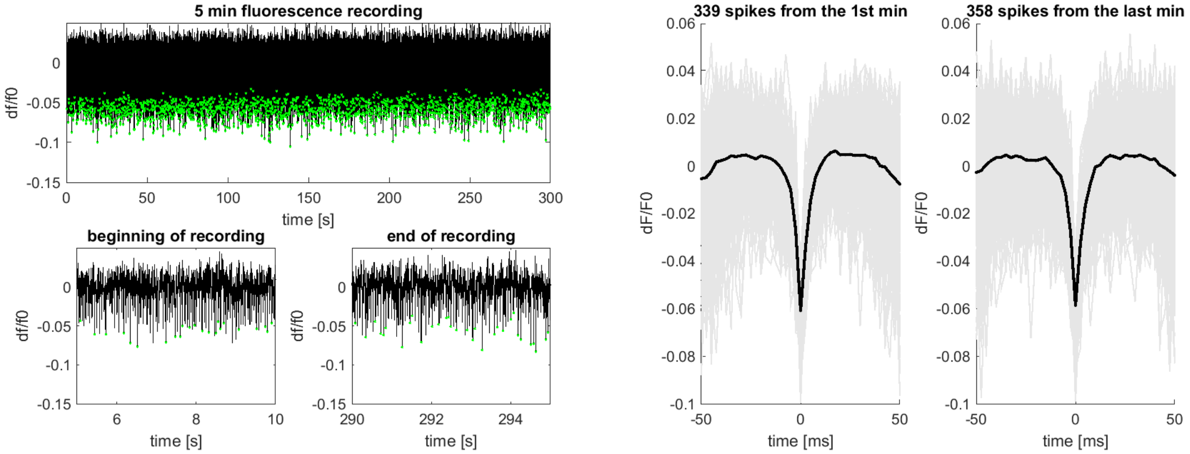

Voltron cell 5 (ΔF/F=7.9, SNR=5.7)

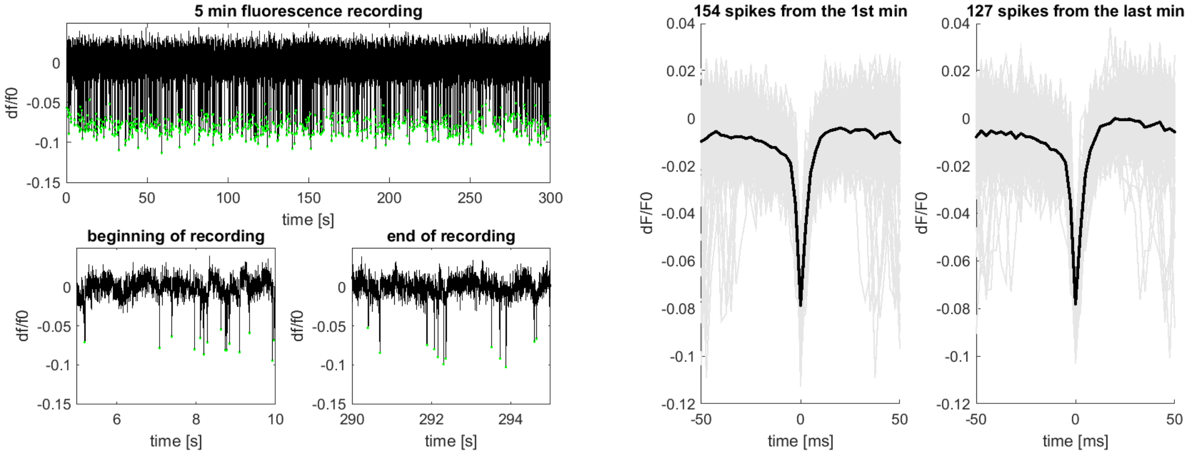

Voltron cell 6 (ΔF/F=8.7, SNR=4.2)

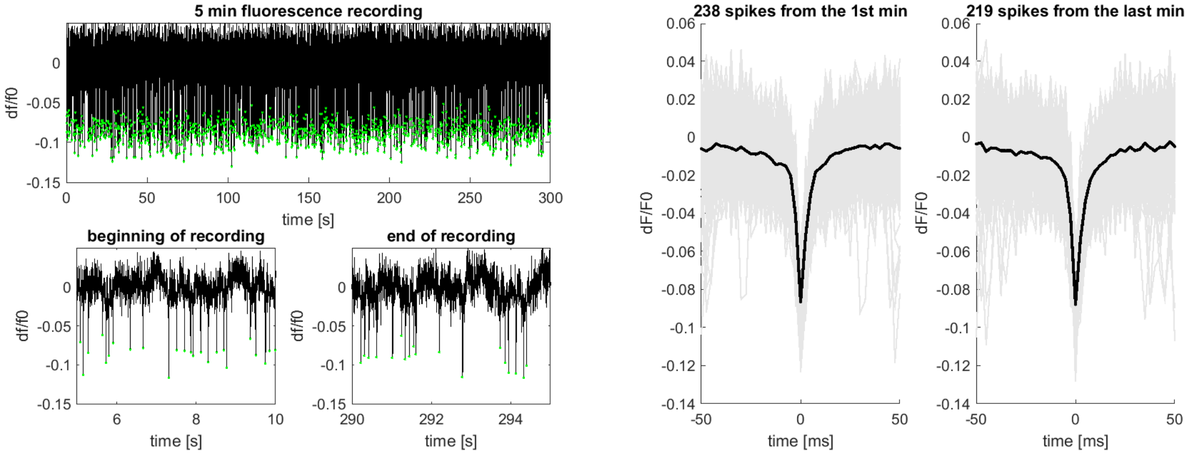

Voltron cell 7 (ΔF/F=5.5, SNR=4.2)

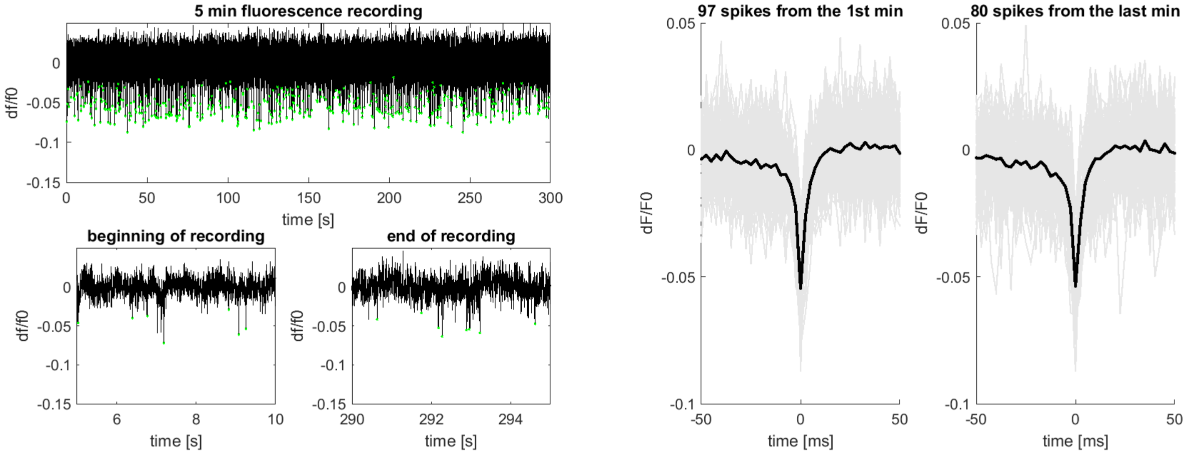

Voltron cell 8 (ΔF/F=8.0, SNR=4.5)

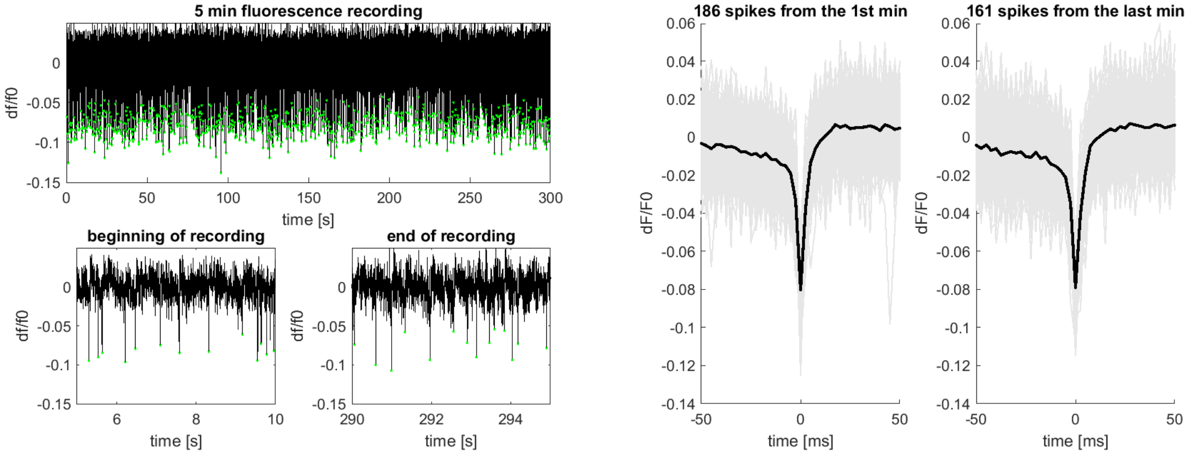

Voltron cell 9 (ΔF/F=6.6, SNR=4.2)

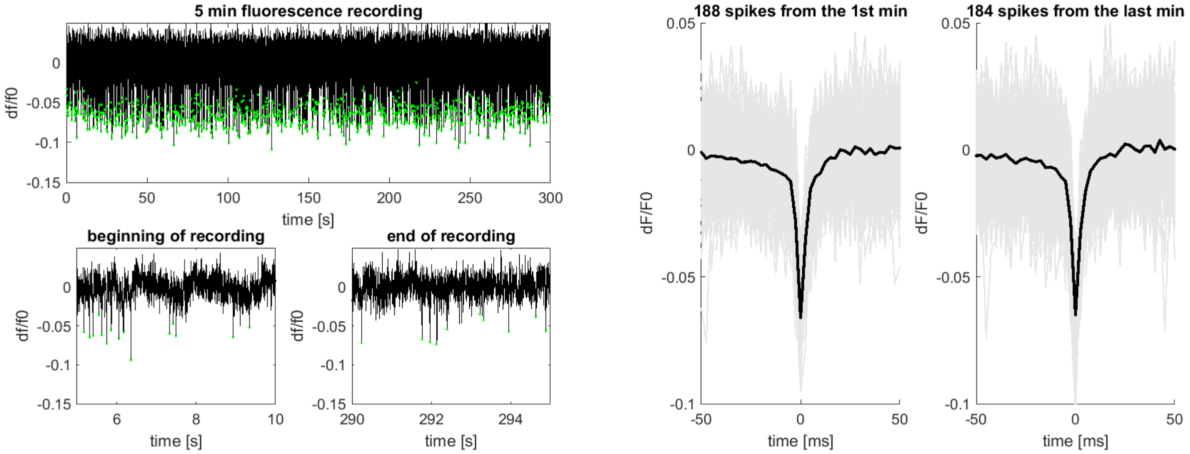

Voltron cell 10 (ΔF/F=5.8, SNR=4.6)

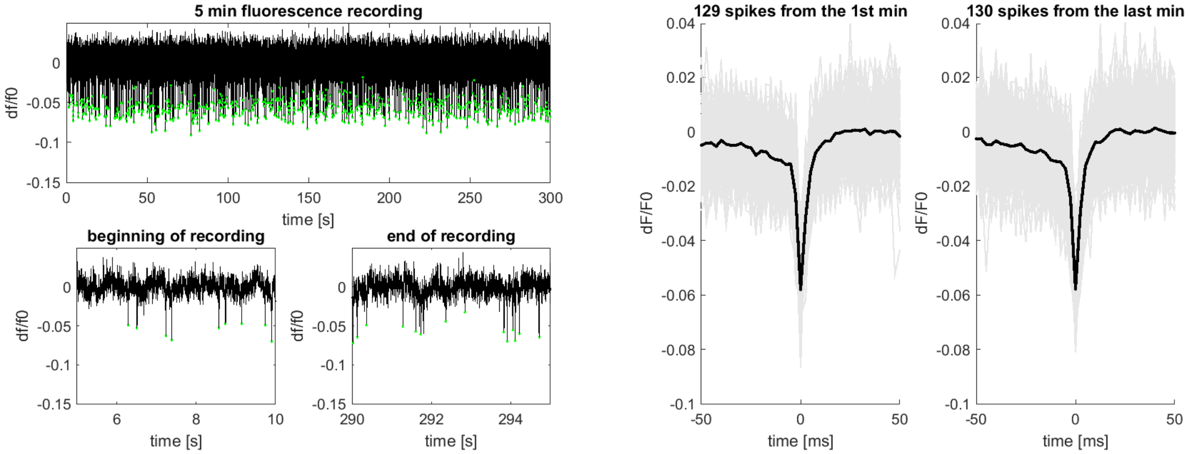

C

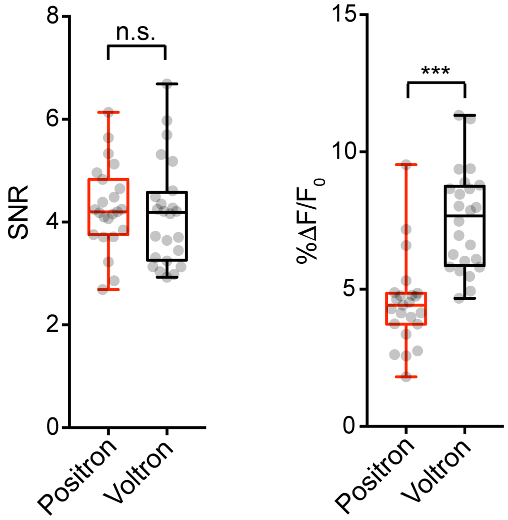

Supplementary Figure 8. Comparison of Positron (red) and Voltron (black) used to image spontaneous activity in olfactory sensory neurons of larval zebrafish. (A) Schematic of preparation imaged and representative image of a labeled olfactory sensory neuron. Scale bar 20 μm. (B) Example fluorescence traces from 10 neurons expressing Positron (red) and 10 neurons expressing Voltron (black). Each panel shows the full 5 minute fluorescence recording (top left) as well as 5 second zooms from the beginning and end of the recording (bottom left). Detected spikes are marked with a green dot. Mean spike waveforms from both the first minute and the last minute of recording are shown on the right. (C) Comparison of SNR and $\Delta F/F_0$ for N = 23 cells for Positron, N = 24 cells for Voltron. Box represents the interquartile range (25–75 percentile), center represents median value, whiskers represent the minimum and maximum of all data. For $\Delta F/F_0$ plot n.s.: non-significant and ***P < 0.001 for two-tailed unpaired t test.

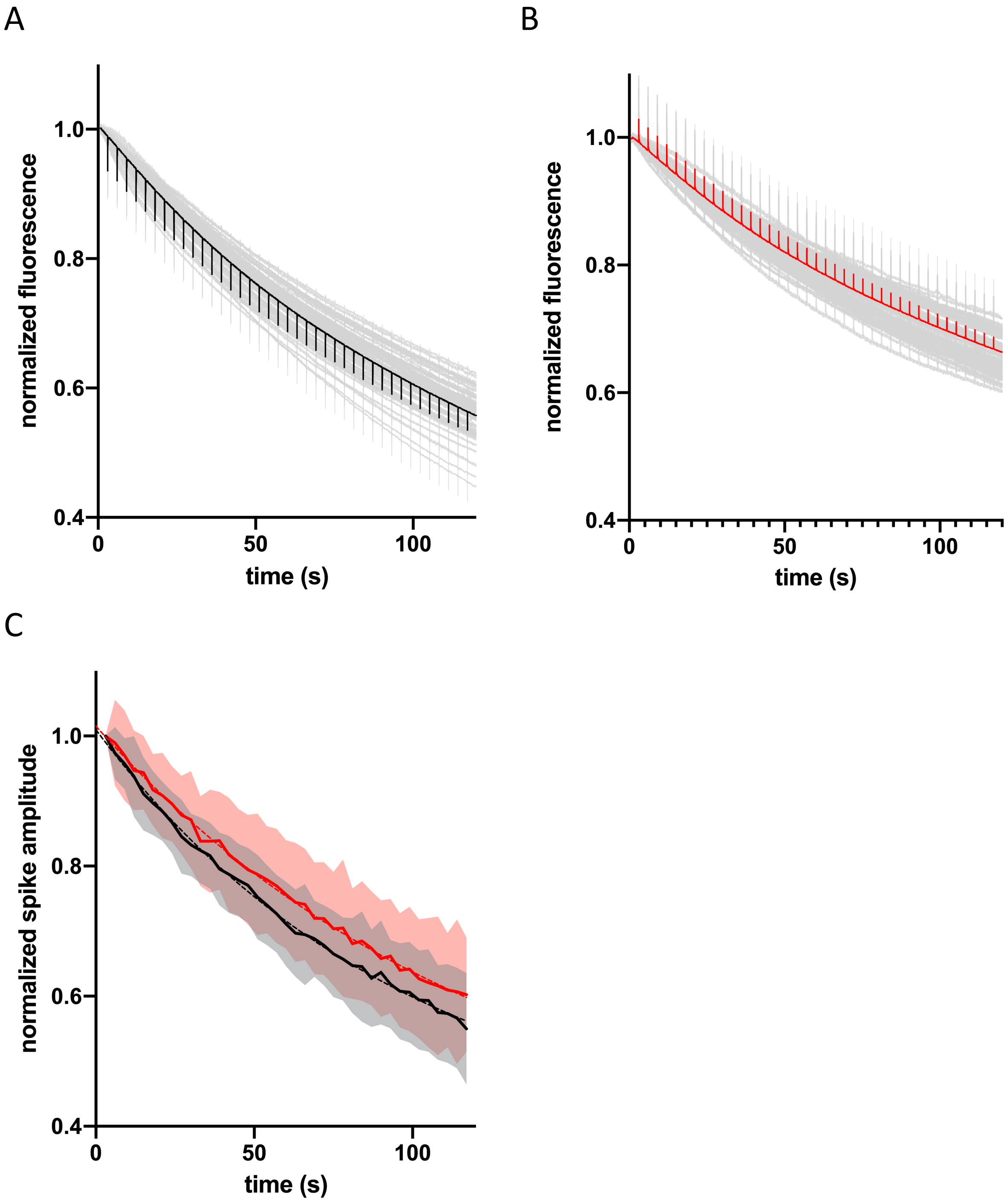

Supplementary Figure 9. Comparison of Voltron (A) and Positron (B) fluorescence decay in neuron cultures with continuous high-intensity illumination and periodic stimulation of action potential firing. Light grey traces represent ROIs from individual neurons, black and red traces represent the mean for Voltron and Positron, respectively. N = 68 cells for Voltron, N = 65 cells for Positron. Three independent transfections for each construct. (C) Comparison of the decay of the normalized spike amplitudes from panels (A) and (B). Solid lines represent the mean values, and light grey and red shaded areas are ± std.

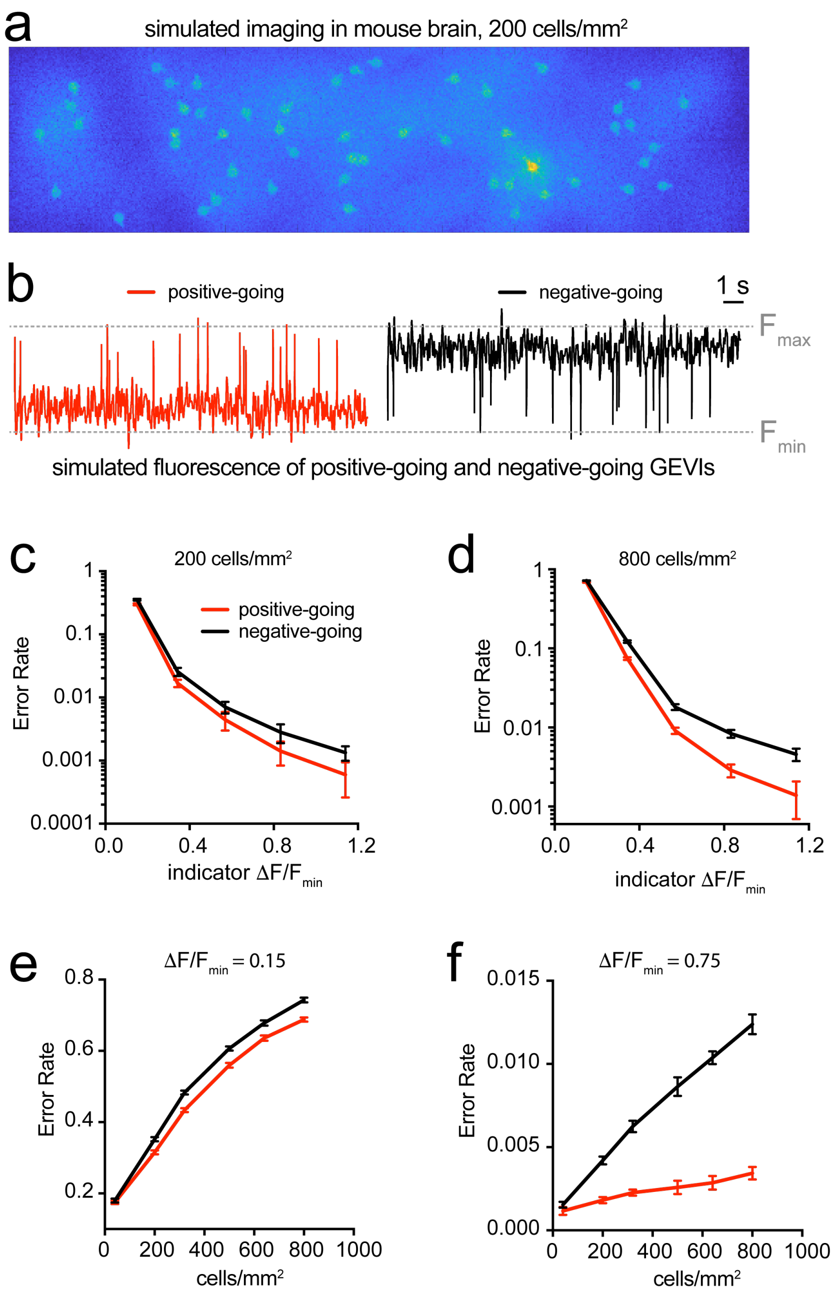

Supplementary Figure 10. Simulations comparing positive- and negative-going indicators. (A) Example frame of simulation produced by adding 50 simulated to a real recording of sparsely-labeled mouse cortex. (B) Example simulated indicator traces with dF/F_{min} of 0.4. We simulated performance of indicators with equal response magnitude (F_{max}-F_{min}) but either positive- or negative- going responses. (C-D). Error rate of spike inference (mean ± SEM) for the two indicators at different values of $\Delta F/F_{min}$, with 200 cells/mm^2 (C) or 800 cells/mm^2 (D). N = 400 simulated cells per condition. (E-F) Error rate of spike inference (mean ± SEM) for the two indicators with different numbers of simulated cells, with $\Delta F/F_{min}$=0.15 (C) and $\Delta F/F_{min}$=0.75 (D).

For $\Delta F/F_{min}$=0.15, N = 125-200 cells per condition. For $\Delta F/F_{min}$=0.75, N = 375-600 cells per condition.

```
Ace2
atggctgacgtggaaaccgagaccggcatgattgcacagtggattgtctttgctattatggctgctgctgctattgcttttggagtggctgtgcactttc
 M  A  D  V  E  T  E  T  G  M  I  A  Q  W  I  V  F  A  I  M  A  A  A  A  I  A  F  G  V  A  V  H  F
Ace2
ggccttcagagctgaagagcgcatactatatcaacattgccatctgcactatcgccgctaccgcttactatgcaatggccgtgaactaccaggacctgac
R  P  S  E  L  K  S  A  Y  Y  I  N  I  A  I  C  T  I  A  A  T  A  Y  Y  A  M  A  V  N  Y  Q  D  L  T
Ace2
                                       PA                                     PD
aatgaatggtgaaaggcaggtggtctacgcaagatatattgactgggtgctgaccacaccactgctcctgctcaacctcatcgtcatgaccaagatgggc
  M  N  G  E  R  Q  V  V  Y  A  R  Y  I  D  W  V  L  T  T  P  L  L  L  L  N  L  I  V  M  T  K  M  G
Ace2
ggagtgatgatttcttgggtcatcggcgcagacattttcatgatcgtgtttggtattctgggcgccttcgaggatgaacacaagttcaaatgggtgtact
 G  V  M  I  S  W  V  I  G  A  D  I  F  M  I  V  F  G  I  L  G  A  F  E  D  E  H  K  F  K  W  V  Y
Ace2
ttatcgctggatgtgtgatgcaggcagtcctgacatacgggatgtataacgccacttggaaagacgatctgaagaaaagccccgagtaccatagctccta
F  I  A  G  C  V  M  Q  A  V  L  T  Y  G  M  Y  N  A  T  W  K  D  D  L  K  K  S  P  E  Y  H  S  S  Y
Ace2
                                                                                                  PR
tgtcagtctgctcgtcttcctgtcaatcctctgggtgttttatcctgtcgtgtgggctttcgggtctggtagtggcgtgctgtccgtcgacaatgtggcc
  V  S  L  L  V  F  L  S  I  L  W  V  F  Y  P  V  V  W  A  F  G  S  G  S  G  V  L  S  V  D  N  V  A
Ace2                                                                                         mNeo...een
attctcatgggaatcctggatgtgctcgctaagccactgtttggaatggggtgcctcattgcccatgagactatcttcaagaagatgctgaggtctctcc
 I  L  M  G  I  L  D  V  L  A  K  P  L  F  G  M  G  C  L  I  A  H  E  T  I  F  K  K  M  L  R  S  L
mNeonGreen
cagcgacacatgagttacacatctttggctccatcaacggtgtggactttgacatggtgggtcagggcaccggcaatccaaatgatggttatgaggagtt
P  A  T  H  E  L  H  I  F  G  S  I  N  G  V  D  F  D  M  V  G  Q  G  T  G  N  P  N  D  G  Y  E  E  L
mNeonGreen
aaacctgaagtccaccaagggtgacctccagttctcccctggattctggtccctcatatcgggtatggcttccatcagtacctgccctaccctgacggg
  N  L  K  S  T  K  G  D  L  Q  F  S  P  W  I  L  V  P  H  I  G  Y  G  F  H  Q  Y  L  P  Y  P  D  G
mNeonGreen
atgtcgcctttccaggccgccatggtagatggctccggataccaagtccatcgcacaatgcagtttgaagatggtgcctcccttactgttaactaccgct
 M  S  P  F  Q  A  A  M  V  D  G  S  G  Y  Q  V  H  R  T  M  Q  F  E  D  G  A  S  L  T  V  N  Y  R
mNeonGreen
acacctacgagggaagccacatcaaaggagaggcccaggtgaaggggactggtttccctgctgacggtcctgtgatgaccaactcgctgaccgctgcgga
Y  T  Y  E  G  S  H  I  K  G  E  A  Q  V  K  G  T  G  F  P  A  D  G  P  V  M  T  N  S  L  T  A  A  D
mNeonGreen
ctggtgcaggtcgaagaagacttaccccaacgacaaaaccatcatcagtacctttaagtggagttacaccactggaaatggcaagcgctacaggagcact
  W  C  R  S  K  K  T  Y  P  N  D  K  T  I  I  S  T  F  K  W  S  Y  T  T  G  N  G  K  R  Y  R  S  T
mNeonGreen
gcgcggaccacctacacctttgccaagccaatggcggctaactatctgaagaaccagccgatgtacgtgttccgtaagacggagctcaagcactccaaga
 A  R  T  T  Y  T  F  A  K  P  M  A  A  N  Y  L  K  N  Q  P  M  Y  V  F  R  K  T  E  L  K  H  S  K
mNeonGreen                                              Kir2.1 membrane trafficking signal
ccgagctcaacttcaaggagtggcaaaaggcctttaccgatgtgatgggcatggacgagctgtacaagaagagcaggatcaccagcgagggcgagtacat
T  E  L  N  F  K  E  W  Q  K  A  F  T  D  V  M  G  M  D  E  L  Y  K  K  S  R  I  T  S  E  G  E  Y  I
Kir2.1 membrane trafficking signal   Kir2.1 ER export signal
cccctggaccagatcgacatcaacgtgttctgctacgagaacgaggtgtaa
  P  L  D  Q  I  D  I  N  V  F  C  Y  E  N  E  V  .
```

Supplementary Figure 11. DNA and amino acid sequence of Ace2_D92N_E199V-mNeonGreen with sequence features annotated. Note the three point mutations that are responsible for the positive fluorescence voltage slope annotated as PA (proton acceptor position), PD (proton donor position), and PR (proton release position).

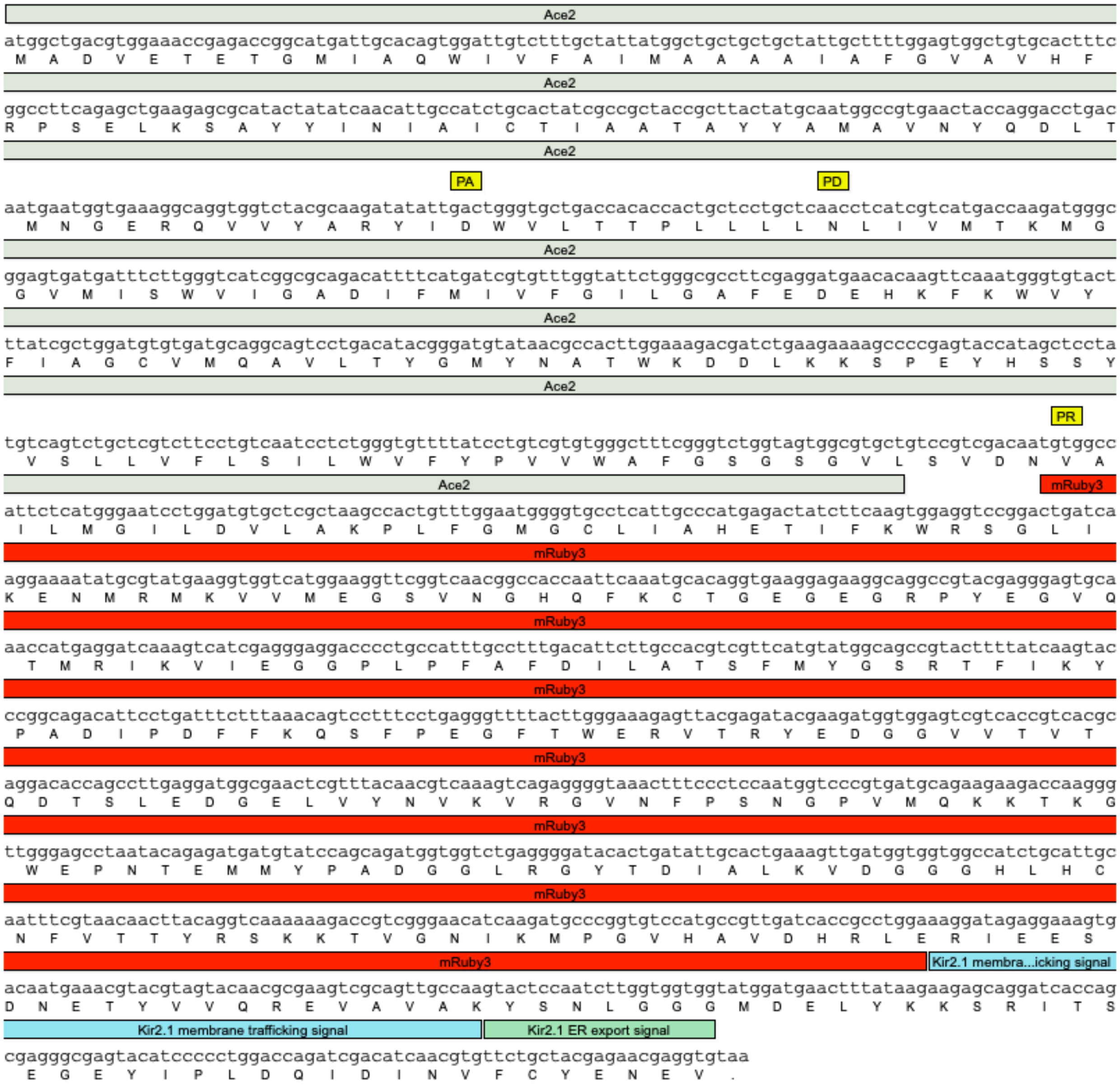

Supplementary Figure 12. DNA and amino acid sequence of Ace2_D92N_E199V-mRuby3 with sequence features annotated. Note the three point mutations that are responsible for the positive fluorescence voltage slope annotated as PA (proton acceptor position), PD (proton donor position), and PR (proton release position).

```
Mac
atgatcgttgatcagttcgaggaggttctcatgaaaacatcacagctcttccctctgccaactgcaacacaatctgcccaacctacccatgttgcacctg
M I V D Q F E E V L M K T S Q L F P L P T A T Q S A Q P T H V A P
Mac
taccgaccgtccttcctgacacaccgatttacgagaccgtcggagatagtggttccaaaactctttgggtagtattcgtgttgatgctcatagcatccgc
V P T V L P D T P I Y E T V G D S G S K T L W V V F V L M L I A S A
Mac
agctttcacggccttgagctggaagatacctgtaaaccggaggttgtaccacgtaatcaccactatcatcacactcacagccgcactgtcttattttgcc
A F T A L S W K I P V N R R L Y H V I T T I I T L T A A L S Y F A
Mac
atggcaaccggccatggagtcgccctcaacaagatagtcatcaggacgcaacacgaccatgtgccggacacctatgaaaccgtatataggcaggtatact
M A T G H G V A L N K I V I R T Q H D H V P D T Y E T V Y R Q V Y
Mac
PA PD
acgcacggtacattgattgggcaataacaaccccattgcttctgctgaatctcgggttgcttgcagggatgtcaggagcacacatttttatggcaattgt
Y A R Y I D W A I T T P L L L L N L G L L A G M S G A H I F M A I V
Mac
tgccgatcttattatggtcttgacgggattgttcgctgcctttggcagtgaagggactcctcaaaaatggggctggtatacgattgcttgcatcgcctac
A D L I M V L T G L F A A F G S E G T P Q K W G W Y T I A C I A Y
Mac
atctttgttgtctggcatctcgtacttaacggcggtgctaacgccagggttaaaggggagaagcttcggtctttttcgttgctatcggagcctacaccc
I F V V W H L V L N G G A N A R V K G E K L R S F F V A I G A Y T
Mac
PR
tcatcttgtggacagcttatcctatcgtttggggcctggccgacggggctcgcaaaattggcgtagacggggtgattattgcctatgcagttctggatgt
L I L W T A Y P I V W G L A D G A R K I G V D G V I I A Y A V L D V
Mac HaloTag
actggctaaaggggtcttcggtgcatggctcctggtcactcatgccaatcttcgcgaatcagacatcggtaccggctttccattcgaccccattatgtg
L A K G V F G A W L L V T H A N L R E S D I G T G F P F D P H Y V
HaloTag
gaagtcctgggcgagcgcatgcactacgtcgatgttggtccgcgcgatggcacccctgtgctgttcctgcacggtaacccgacctcctcctacgtgtggc
E V L G E R M H Y V D V G P R D G T P V L F L H G N P T S S Y V W
HaloTag
gcaacatcatcccgcatgttgcaccgacccatcgctgcattgctccagacctgatcggtatgggcaaatccgacaaaccagacctgggttatttcttcga
R N I I P H V A P T H R C I A P D L I G M G K S D K P D L G Y F F D
HaloTag
cgaccacgtccgcttcatggatgccttcatcgaagccctgggtctggaagaggtcgtcctggtcattcacgactggggctccgctctgggtttccactgg
D H V R F M D A F I E A L G L E E V V L V I H D W G S A L G F H W
HaloTag
gccaagcgcaatccagagcgcgtcaaaggtattgcatttatggagttcatccgccctatcccgacctgggacgaatggccagaatttgcccgcgagacct
A K R N P E R V K G I A F M E F I R P I P T W D E W P E F A R E T
HaloTag
tccaggccttccgcaccaccgacgtcggccgcaagctgatcatcgatcagaacgtttttatcgagggtacgctgccgatgggtgtcgtccgcccgctgac
F Q A F R T T D V G R K L I I D Q N V F I E G T L P M G V V R P L T
HaloTag
tgaagtcgagatggaccattaccgcgagccgttcctgaatcctgttgaccgcgagccactgtggcgcttcccaaacgagctgccaatcgccggtgagcca
E V E M D H Y R E P F L N P V D R E P L W R F P N E L P I A G E P
HaloTag
gcgaacatcgtcgcgctggtcgaagaatacatggactggctgcaccagtcccctgtcccgaagctgctgttctggggcaccccaggcgttctgatcccac
A N I V A L V E E Y M D W L H Q S P V P K L L F W G T P G V L I P
HaloTag
cggccgaagccgctcgcctggccaaaagcctgcctaactgcaaggctgtggacatcggcccgggtctgaatctgctgcaagaagacaacccggacctgat
P A E A A R L A K S L P N C K A V D I G P G L N L L Q E D N P D L I
HaloTag Kir2.1 membrane trafficking signal
cggcagcgagatcgcgcgctggctgtcgacgctcgagatttccggcgagccaaccactaagagcaggatcaccagcgagggcgagtacatcccctggac
G S E I A R W L S T L E I S G E P T T K S R I T S E G E Y I P L D
Kir2.1 membran...ficking signal Kir2.1 ER export signal
cagatcgacatcaacgtgttctgctacgagaacgaggtgtaa
Q I D I N V F C Y E N E V .
```

Supplementary Figure 13. DNA and amino acid sequence of Mac_Q139D_D150N_E258V-HaloTag with sequence features annotated. Note the three point mutations that are responsible for the positive fluorescence voltage slope annotated as PA (proton acceptor position), PD (proton donor position), and PR (proton release position).

```
QuasAr3
atggtatctatcgcacttcaagccggttatgatctcctgggagatgggcggcctgaaacactttggctcgggatcggcactttgcttatgctcattggca
 M  V  S  I  A  L  Q  A  G  Y  D  L  L  G  D  G  R  P  E  T  L  W  L  G  I  G  T  L  L  M  L  I  G
QuasAr3
cgttttattttctggtacgaggatggggggtcactgacaaagatgcaagggagtactacgcagtgacgattcttgtaagtggcatcgccagtgctgctta
T  F  Y  F  L  V  R  G  W  G  V  T  D  K  D  A  R  E  Y  Y  A  V  T  I  L  V  S  G  I  A  S  A  A  Y
QuasAr3
PA
cctgtcaatgtttttcggaatcggcctcacagaggtaagcgttggtggggaaatgctcgacatttattatgctaggtacgccgactggttgtttacgaca
  L  S  M  F  F  G  I  G  L  T  E  V  S  V  G  G  E  M  L  D  I  Y  Y  A  R  Y  A  D  W  L  F  T  T
QuasAr3
PD
ccactcctgctgctcaacttggccttgttggctaaggtagatagggtcactatcggcaccctggtaggagtggatgcactgatgattgtaacggggctga
 P  L  L  L  L  N  L  A  L  L  A  K  V  D  R  V  T  I  G  T  L  V  G  V  D  A  L  M  I  V  T  G  L
QuasAr3
ttggagccttgagtcatactgcaatcgcaaggtactcatggtggctgttctctacaatatgtatgattgtggtgctctatgtgttggctacgtcacttcg
I  G  A  L  S  H  T  A  I  A  R  Y  S  W  W  L  F  S  T  I  C  M  I  V  V  L  Y  V  L  A  T  S  L  R
QuasAr3
gtccgctgcacgcgaaagaggacctgaagtcgccagtacttttaataccctgacagctttggtgctcgtactgtggaccgcatatcccattctttggatt
  S  A  A  R  E  R  G  P  E  V  A  S  T  F  N  T  L  T  A  L  V  L  V  L  W  T  A  Y  P  I  L  W  I
QuasAr3
PR
attggtacggagggagcaggtgttgttggactcggtatcgtgaccctcctcttcatggttctggacgtaactgcaaaggttgggtttggcttcatccttc
 I  G  T  E  G  A  G  V  V  G  L  G  I  V  T  L  L  F  M  V  L  D  V  T  A  K  V  G  F  G  F  I  L
QuasAr3 | HaloTag
tcaggagtcgcgcaatattgggagacactgagatcggtaccggctttccattcgacccccattatgtggaagtcctgggcgagcgcatgcactacgtcga
L  R  S  R  A  I  L  G  D  T  E  I  G  T  G  F  P  F  D  P  H  Y  V  E  V  L  G  E  R  M  H  Y  V  D
HaloTag
tgttggtccgcgcgatggcacccctgtgctgttcctgcacggtaacccgacctcctcctacgtgtggcgcaacatcatcccgcatgttgcaccgacccat
  V  G  P  R  D  G  T  P  V  L  F  L  H  G  N  P  T  S  S  Y  V  W  R  N  I  I  P  H  V  A  P  T  H
HaloTag
cgctgcattgctccagacctgatcggtatgggcaaatccgacaaaccagacctgggttatttcttcgacgaccacgtccgcttcatggatgccttcatcg
 R  C  I  A  P  D  L  I  G  M  G  K  S  D  K  P  D  L  G  Y  F  F  D  D  H  V  R  F  M  D  A  F  I
HaloTag
aagccctgggtctggaagaggtcgtcctggtcattcacgactggggctccgctctgggtttccactgggccaagcgcaatccagagcgcgtcaaaggtat
E  A  L  G  L  E  E  V  V  L  V  I  H  D  W  G  S  A  L  G  F  H  W  A  K  R  N  P  E  R  V  K  G  I
HaloTag
tgcatttatggagttcatccgccctatcccgacctgggacgaatggccagaatttgcccgcgagaccttccaggccttccgcaccaccgacgtcggccgc
  A  F  M  E  F  I  R  P  I  P  T  W  D  E  W  P  E  F  A  R  E  T  F  Q  A  F  R  T  T  D  V  G  R
HaloTag
aagctgatcatcgatcagaacgtttttatcgagggtacgctgccgatgggtgtcgtccgcccgctgactgaagtcgagatggaccattaccgcgagccgt
 K  L  I  I  D  Q  N  V  F  I  E  G  T  L  P  M  G  V  V  R  P  L  T  E  V  E  M  D  H  Y  R  E  P
HaloTag
tcctgaatcctgttgaccgcgagccactgtggcgcttcccaaacgagctgccaatcgccggtgagccagcgaacatcgtcgcgctggtcgaagaatacat
F  L  N  P  V  D  R  E  P  L  W  R  F  P  N  E  L  P  I  A  G  E  P  A  N  I  V  A  L  V  E  E  Y  M
HaloTag
ggactggctgcaccagtcccctgtcccgaagctgctgttctggggcaccccaggcgttctgatcccaccggccgaagccgctcgcctggccaaaagcctg
  D  W  L  H  Q  S  P  V  P  K  L  L  F  W  G  T  P  G  V  L  I  P  P  A  E  A  A  R  L  A  K  S  L
HaloTag
cctaactgcaaggctgtggacatcggcccgggtctgaatctgctgcaagaagacaacccggacctgatcggcagcgagatcgcgcgctggctgtcgacgc
 P  N  C  K  A  V  D  I  G  P  G  L  N  L  L  Q  E  D  N  P  D  L  I  G  S  E  I  A  R  W  L  S  T
HaloTag | Kir2.1 membrane trafficking signal | Kir2.1 ER export signal
tcgagatttccggcgagccaaccactaagagcaggatcaccagcgagggcgagtacatcccccctggaccagatcgacatcaacgtgttctgctacgagaa
L  E  I  S  G  E  P  T  T  K  S  R  I  T  S  E  G  E  Y  I  P  L  D  Q  I  D  I  N  V  F  C  Y  E  N
Kir2....ignal
cgaggtgtaa
  E  V  .
```

Supplementary Figure 14. DNA and amino acid sequence of QuasAr3_Q95D_H106N_E214V-HaloTag with sequence features annotated. Note the three point mutations that are responsible for the positive fluorescence voltage slope annotated as PA (proton acceptor position), PD (proton donor position), and PR (proton release position).

```
Ace1
atgagcaatccaaatccctttcagactactcttggcacggacgcccagtgggttgtctttgccgtaatggcactggcagccatagtgtttagtatagcag
 M  S  N  P  N  P  F  Q  T  T  L  G  T  D  A  Q  W  V  V  F  A  V  M  A  L  A  A  I  V  F  S  I  A
Ace1
ttcagtttcggccactcccgctgcgcctcacttactacgtcaacattgctatttgtacaatagccgcaacagcttactatgccatggccgttaatggcgg
V  Q  F  R  P  L  P  L  R  L  T  Y  Y  V  N  I  A  I  C  T  I  A  A  T  A  Y  Y  A  M  A  V  N  G  G
Ace1
                                                                                    PA                                 PD
agacaataaaccgacagctgggacgggggcagacgaacgccaggttatttacgctcgctatatcgactgggtgtttaccacaccactcctcttgcttaat
  D  N  K  P  T  A  G  T  G  A  D  E  R  Q  V  I  Y  A  R  Y  I  D  W  V  F  T  T  P  L  L  L  L  N
Ace1
ctggtgttgttgactaacatgccagccactatgatcgcttggattatgggagcagacatcgctatgatcgcttttggaatcatcggtgctttcaccgtcg
 L  V  L  L  T  N  M  P  A  T  M  I  A  W  I  M  G  A  D  I  A  M  I  A  F  G  I  I  G  A  F  T  V
Ace1
gctcttacaagtggtttacttcgtagtggggtgcataatgctcgcagtccttgcttggggatgataaaccccatattcaaagaggaattgcagaaaca
G  S  Y  K  W  F  Y  F  V  V  G  C  I  M  L  A  V  L  A  W  G  M  I  N  P  I  F  K  E  E  L  Q  K  H
Ace1
caaagagtacacaggggcctacacgactctgctcatatatctcatagtcttgtgggttatatatccaattgtctggggtctcggagcaggcgggcacatt
  K  E  Y  T  G  A  Y  T  T  L  L  I  Y  L  I  V  L  W  V  I  Y  P  I  V  W  G  L  G  A  G  G  H  I
Ace1
             PR
ataggagtggatgtagtgattatcgcaatgggtgttctggacctgcttgccaagccgctttacgctataggggttctcataacggttgaggttgtttacg
 I  G  V  D  V  V  I  I  A  M  G  V  L  D  L  L  A  K  P  L  Y  A  I  G  V  L  I  T  V  E  V  V  Y
Ace1 HaloTag
gaaagatcggtaccggctttccattcgaccccattatgtggaagtcctgggcgagcgcatgcactacgtcgatgttggtccgcgcgatggcacccctgt
G  K  I  G  T  G  F  P  F  D  P  H  Y  V  E  V  L  G  E  R  M  H  Y  V  D  V  G  P  R  D  G  T  P  V
HaloTag
gctgttcctgcacggtaacccgacctcctcctacgtgtggcgcaacatcatcccgcatgttgcaccgacccatcgctgcattgctccagacctgatcggt
  L  F  L  H  G  N  P  T  S  S  Y  V  W  R  N  I  I  P  H  V  A  P  T  H  R  C  I  A  P  D  L  I  G
HaloTag
atgggcaaatccgacaaaccagacctgggttatttcttcgacgaccacgtccgcttcatggatgccttcatcgaagccctgggtctggaagaggtcgtcc
 M  G  K  S  D  K  P  D  L  G  Y  F  F  D  D  H  V  R  F  M  D  A  F  I  E  A  L  G  L  E  E  V  V
HaloTag
tggtcattcacgactggggctccgctctgggtttccactgggccaagcgcaatccagagcgcgtcaaaggtattgcatttatggagttcatccgccctat
L  V  I  H  D  W  G  S  A  L  G  F  H  W  A  K  R  N  P  E  R  V  K  G  I  A  F  M  E  F  I  R  P  I
HaloTag
cccgacctgggacgaatggccagaatttgcccgcgagaccttccaggccttccgcaccaccgacgtcggccgcaagctgatcatcgatcagaacgttttt
  P  T  W  D  E  W  P  E  F  A  R  E  T  F  Q  A  F  R  T  T  D  V  G  R  K  L  I  I  D  Q  N  V  F
HaloTag
atcgagggtacgctgccgatgggtgtcgtccgcccgctgactgaagtcgagatggaccattaccgcgagccgttcctgaatcctgttgaccgcgagccac
 I  E  G  T  L  P  M  G  V  V  R  P  L  T  E  V  E  M  D  H  Y  R  E  P  F  L  N  P  V  D  R  E  P
HaloTag
tgtggcgcttcccaaacgagctgccaatcgccggtgagccagcgaacatcgtcgcgctggtcgaagaatacatggactggctgcaccagtcccctgtccc
L  W  R  F  P  N  E  L  P  I  A  G  E  P  A  N  I  V  A  L  V  E  E  Y  M  D  W  L  H  Q  S  P  V  P
HaloTag
gaagctgctgttctggggcaccccaggcgttctgatcccaccggccgaagccgctcgcctggccaaaagcctgcctaactgcaaggctgtggacatcggc
  K  L  L  F  W  G  T  P  G  V  L  I  P  P  A  E  A  A  R  L  A  K  S  L  P  N  C  K  A  V  D  I  G
HaloTag
ccgggtctgaatctgctgcaagaagacaacccggacctgatcggcagcgagatcgcgcgctggctgtcgacgctcgagatttccggcgagccaaccacta
 P  G  L  N  L  L  Q  E  D  N  P  D  L  I  G  S  E  I  A  R  W  L  S  T  L  E  I  S  G  E  P  T  T
Kir2.1 membrane trafficking signal   Kir2.1 ER export signal
agagcaggatcaccagcgagggcgagtacatcccctggaccagatcgacatcaacgtgttctgctacgagaacgaggtgtaa
K  S  R  I  T  S  E  G  E  Y  I  P  L  D  Q  I  D  I  N  V  F  C  Y  E  N  E  V  .
```

Supplementary Figure 15. DNA and amino acid sequence of Ace1_Q89D_D100N_E206V-HaloTag with sequence features annotated. Note the three point mutations that are responsible for the positive fluorescence voltage slope annotated as PA (proton acceptor position), PD (proton donor position), and PR (proton release position).

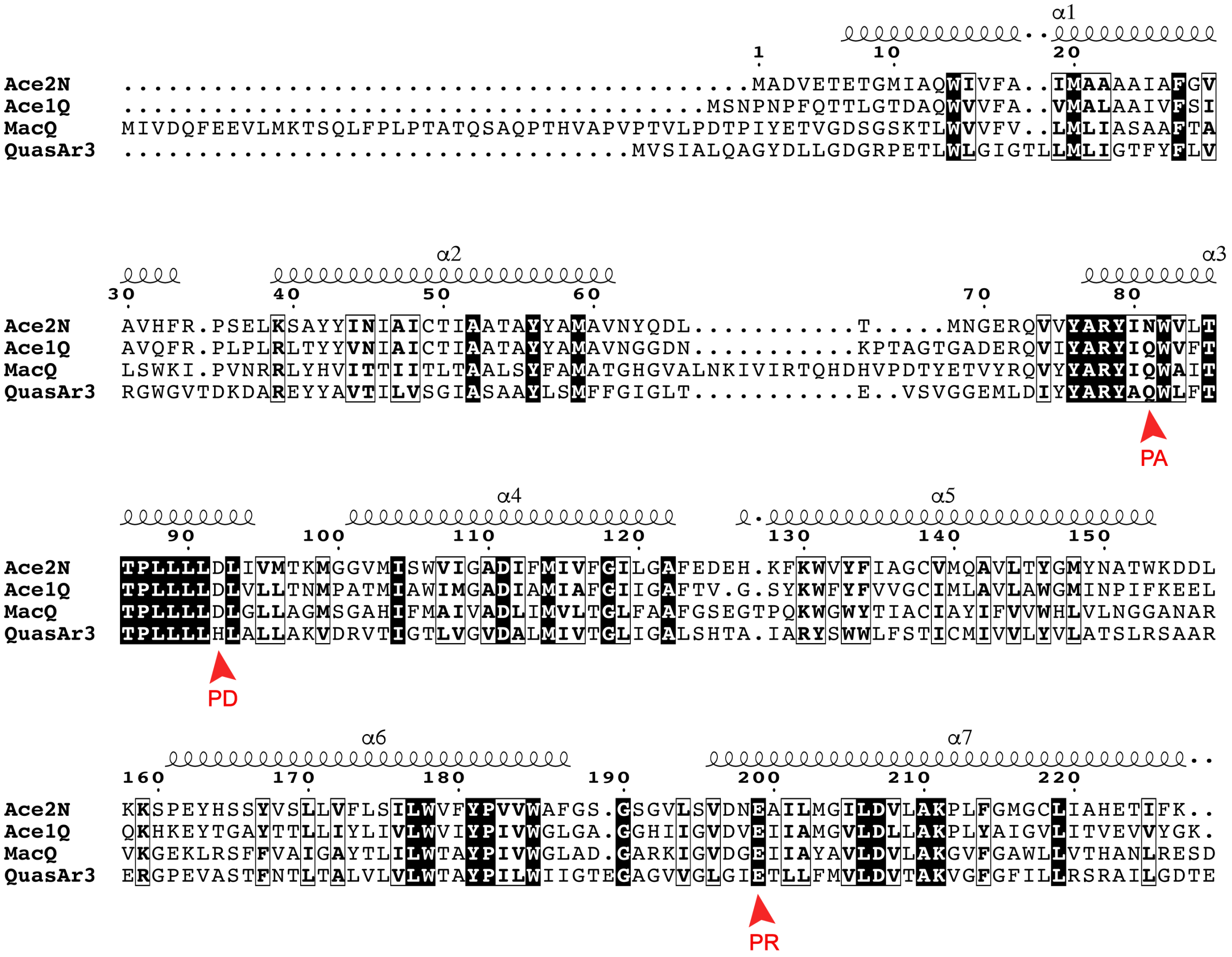

Supplementary Figure 16. Sequence alignment of the four microbial rhodopsins (Ace2N, Ace1Q, MacQ, and QuasAr3) used to construct eFRET GEVI indicators. The proton acceptor (PA), proton donor (PD), and proton release (PR) residues are labeled with red arrows.

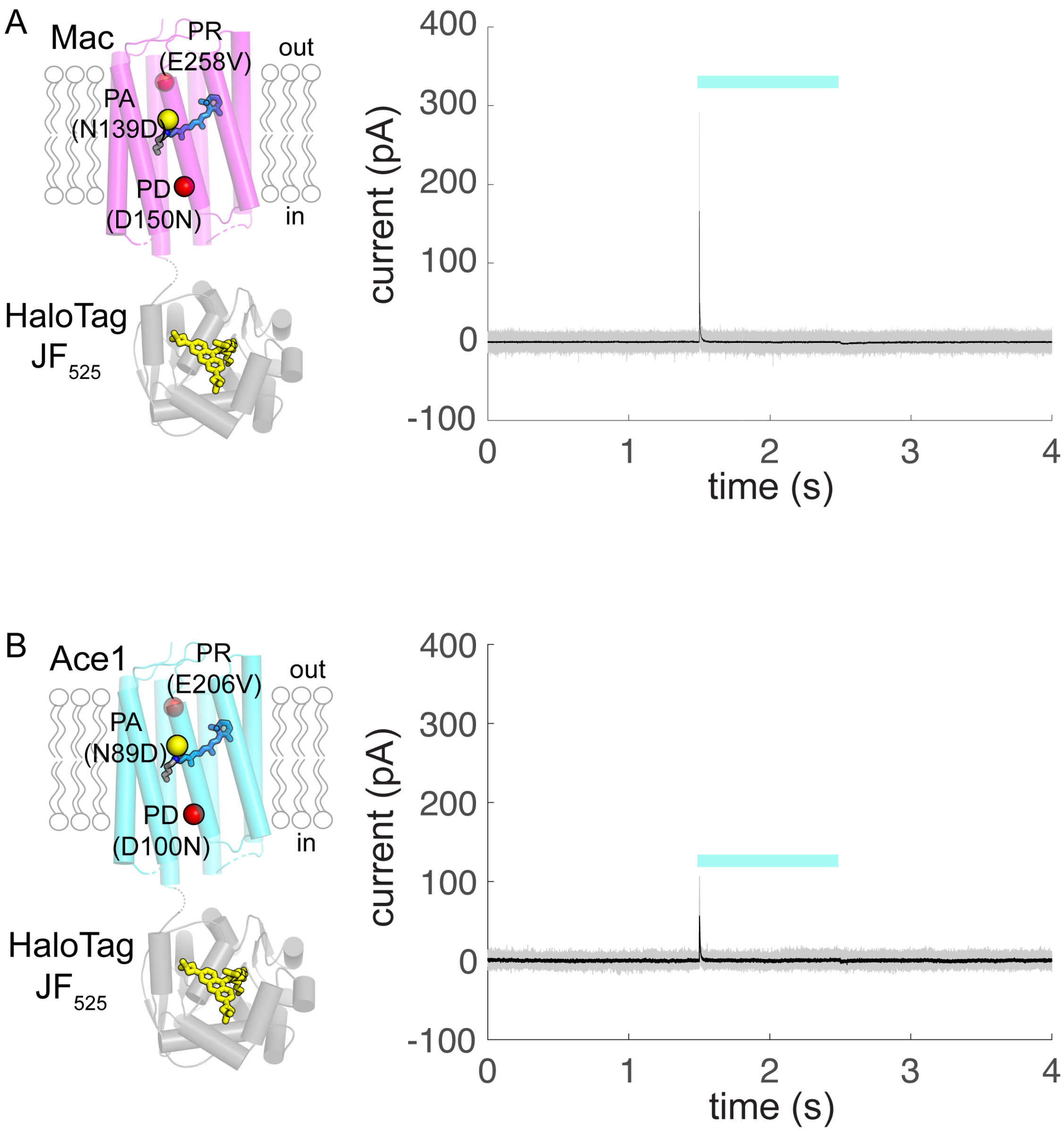

Supplementary Figure 17. Photocurrent measurements for (A) Mac_Q139D_D150N_E258V-HaloTag and (B) Ace1_Q89D_D100N_E206V-HaloTag. Both constructs showed transient outward currents and no steady state photocurrent (0.0 ± 1.7 pA, and –0.6 ± 1.4 pA (mean ± std) for Mac_Q139D_D150N_E258V-HaloTag (N = 3 cells) and Ace1_Q89D_D100N_E206V-HaloTag (N = 4 cells) respectively). Light used was blue/green (508 nm – 522 nm) at an irradiance of 70 mW/mm^2 at the specimen plane. Gray traces: individual current recordings. Black trace: Average of individual current recordings.

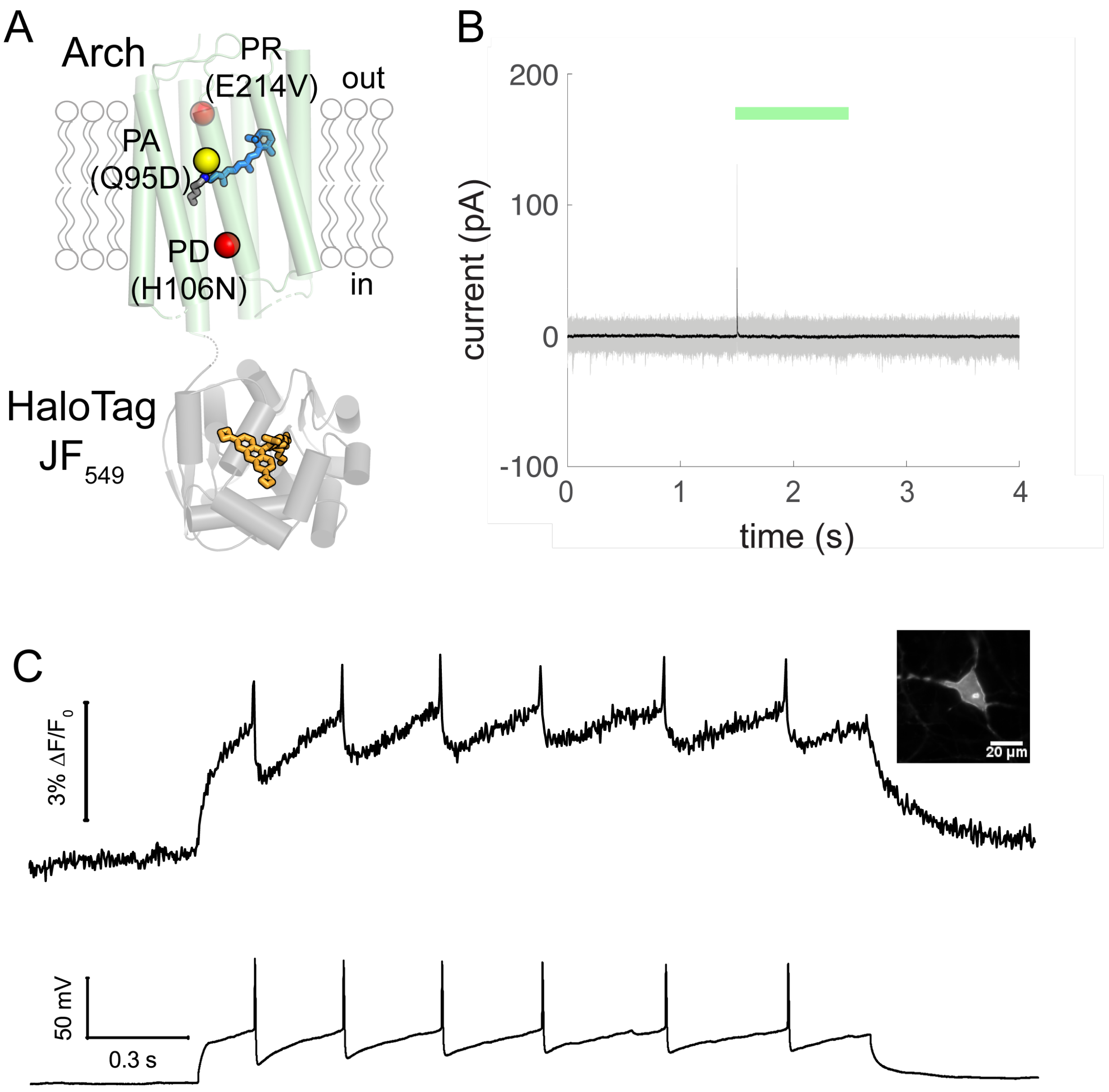

Supplementary Figure 18. (A) Schematic representation of QuasAr3_Q95D_H106N_E214V-HaloTag labeled with JF_{549} dye. Yellow and red spheres are mutations compared to QuasAr3 sequence. (B) Photocurrent measurements for QuasAr3_Q95D_H106N_E214V-HaloTag. QuasAr3_Q95D_H106N_E214V-HaloTag has a transient outward current and no steady state photocurrent (-0.8 ± 3 pA, (mean ± std) (N = 4 cells)). Light used was green (540 nm – 570 nm) at an irradiance of 70 mW/mm^2 at the specimen plane. Gray traces: individual current recordings. Black trace: Average of individual current recordings. (C) Simultaneous recording of fluorescence (recorded at 400 Hz, top) and membrane potential (bottom) in response to current injection of a neuron expressing QuasAr3_Q95D_H106N_E214V-HaloTag-ST and labeled with JF_{549}. Inset: Fluorescence image of patched neuron. Representative of $N \geq 3$ cells.

Supplementary Table1. Positron and Voltron kinetics in primary rat neuron cultures

	Activation (-70 mV to 30 mV)			Deactivation (30 mV to -70 mV)		
	τ_{fast} (ms)	τ_{slow} (ms)	% fast	τ_{fast} (ms)	τ_{slow} (ms)	% fast
Positron-JF_{525}	0.63 ± 0.08	19 ± 6	85 ± 6	0.64 ± 0.10	37 ± 4	90 ± 2
Voltron-JF_{525}	0.64 ± 0.09	4.1 ± 0.6	61 ± 4	0.78 ± 0.12	3.9 ± 0.2	55 ± 7

Neurons expressing Positron were imaged at 3.2 kHz during whole cell voltage clamp as detailed in the methods section. Fluorescence traces were fit using a double exponential function (Supplementary Fig. 4). %fast is the percentage of fluorescence change attributed to the fast-changing component of the bi-exponential fit to the fluorescence change. The remainder is attributed to the slow-changing component. Errors are s.e.m. N = 7 cells for Positron and Voltron measurements are from ref.1

Supplementary References

1. Abdelfattah, A. S. *et al.* Bright and photostable chemigenetic indicators for extended in vivo voltage imaging. *Science.* **365**, 699–704 (2019).
