# Supplementary material for: A general approach to engineer positive-going eFRET voltage indicators
Source: Nat Commun. 2020 Jul 10;11:3444. doi: 10.1038/s41467-020-17322-1 (PMC7351947; doi:10.1038/s41467-020-17322-1)
Supplement: Supplementary file 2 — Reporting Summary [file 41467_2020_17322_MOESM2_ESM.pdf]

nature research

Corresponding author(s): Eric R. Schreiter and Ahmed S. Abdelfattah

Last updated by author(s): May 27, 2020

Reporting Summary

Nature Research wishes to improve the reproducibility of the work that we publish. This form provides structure for consistency and transparency in reporting. For further information on Nature Research policies, see Authors & Referees and the Editorial Policy Checklist .

Statistics

For all statistical analyses, confirm that the following items are present in the figure legend, table legend, main text, or Methods section.

n/a	Confirmed	
☐	☒	The exact sample size (n) for each experimental group/condition, given as a discrete number and unit of measurement
☐	☒	A statement on whether measurements were taken from distinct samples or whether the same sample was measured repeatedly
☐	☒	The statistical test(s) used AND whether they are one- or two-sided<br>*Only common tests should be described solely by name; describe more complex techniques in the Methods section.*
☒	☐	A description of all covariates tested
☒	☐	A description of any assumptions or corrections, such as tests of normality and adjustment for multiple comparisons
☐	☒	A full description of the statistical parameters including central tendency (e.g. means) or other basic estimates (e.g. regression coefficient) AND variation (e.g. standard deviation) or associated estimates of uncertainty (e.g. confidence intervals)
☐	☒	For null hypothesis testing, the test statistic (e.g. F, t, r) with confidence intervals, effect sizes, degrees of freedom and P value noted<br>*Give P values as exact values whenever suitable.*
☒	☐	For Bayesian analysis, information on the choice of priors and Markov chain Monte Carlo settings
☒	☐	For hierarchical and complex designs, identification of the appropriate level for tests and full reporting of outcomes
☒	☐	Estimates of effect sizes (e.g. Cohen's d, Pearson's r), indicating how they were calculated

Our web collection on statistics for biologists contains articles on many of the points above.

Software and code

Policy information about availability of computer code

Data collection	matlab (2018b), imageJ (1.52j), and NIS-Elements (2018)
Data analysis	matlab (2018b), imageJ (1.52j), Prism 7, Origin 2016

For manuscripts utilizing custom algorithms or software that are central to the research but not yet described in published literature, software must be made available to editors/reviewers. We strongly encourage code deposition in a community repository (e.g. GitHub). See the Nature Research guidelines for submitting code & software for further information.

Data

Policy information about availability of data

All manuscripts must include a data availability statement. This statement should provide the following information, where applicable:

- Accession codes, unique identifiers, or web links for publicly available datasets
- A list of figures that have associated raw data
- A description of any restrictions on data availability

All DNA and protein sequences described have been deposited at Addgene (https://www.addgene.org/browse/article/28203791/). Source data from experiments in this study are available from the authors upon reasonable request.

Field-specific reporting

Please select the one below that is the best fit for your research. If you are not sure, read the appropriate sections before making your selection.

☒ Life sciences ☐ Behavioural & social sciences ☐ Ecological, evolutionary & environmental sciences

For a reference copy of the document with all sections, see nature.com/documents/nr-reporting-summary-flat.pdf

Life sciences study design

All studies must disclose on these points even when the disclosure is negative.

Sample size	No specific sample size calculations were performed. Generally, sample size was the maximum possible within reasonable time and resource allocation limits.
Data exclusions	No data were excluded.
Replication	Multiple experimental replicates were performed for each type of measurement as described in the manuscript. All attempts to replicate the data were successful.
Randomization	Data collection for different samples was interleaved to avoid systematic biases over time.
Blinding	No blinding was performed. The study does not contain experiments whose outcome is susceptible to human suggestion.

Reporting for specific materials, systems and methods

We require information from authors about some types of materials, experimental systems and methods used in many studies. Here, indicate whether each material, system or method listed is relevant to your study. If you are not sure if a list item applies to your research, read the appropriate section before selecting a response.

Materials & experimental systems

n/a	Involved in the study	
☒	☐	Antibodies
☐	☒	Eukaryotic cell lines
☒	☐	Palaeontology
☐	☒	Animals and other organisms
☒	☐	Human research participants
☒	☐	Clinical data

Methods

n/a	Involved in the study	
☒	☐	ChIP-seq
☒	☐	Flow cytometry
☒	☐	MRI-based neuroimaging

Eukaryotic cell lines

Policy information about cell lines

Cell line source(s)	Spiking HEK 293 cells from Adam Cohen lab (Harvard)
Authentication	No authentication was performed.
Mycoplasma contamination	Cell cultures were tested and determined to be negative for mycoplasma contamination.
Commonly misidentified lines (See ICLAC register)	No commonly misidentified cell lines were used in the study.

Animals and other organisms

Policy information about studies involving animals; ARRIVE guidelines recommended for reporting animal research

Laboratory animals	Rattus norvegicus (rat), Sprague-Dawley, 0 days, both males and females<br>Danio rerio (zebrafish), Casper, 3 dpf, both males and females
Wild animals	None
Field-collected samples	None
Ethics oversight	All procedures involving animals were conducted in accordance with protocols approved by the Howard Hughes Medical Institute (HHMI) Janelia Research Campus Institutional Animal Care and Use Committee and Institutional Biosafety Committee.

Note that full information on the approval of the study protocol must also be provided in the manuscript.
